# Structure Determination, Mechanical Properties, Thermal Stability of Co_2_MoB_4_ and Fe_2_MoB_4_

**DOI:** 10.3390/ma15093031

**Published:** 2022-04-21

**Authors:** Shijing Zhao, Wenju Zhou, Xiaojun Xiang, Xuyan Cao, Ning Chen, Weifeng Chen, Xiaohui Yu, Bingmin Yan, Huiyang Gou

**Affiliations:** 1 Center for High Pressure Science and Technology Advanced Research, Beijing 100094, China; shijing.zhao@hpstar.ac.cn (S.Z.); wenju.zhou@hpstar.ac.cn (W.Z.); xuyan.cao@hpstar.ac.cn (X.C.); 2 Beijing National Laboratory for Condensed Matter Physics, Institute of Physics, Chinese Academy of Sciences, Beijing 100190, China; xiang_xiaojun@sina.com (X.X.); yuxh@iphy.ac.cn (X.Y.); 3 Canadian Light Source, Saskatoon, SK S7N 2V3, Canada; ning.chen@lightsource.ca (N.C.); weifeng.chen@lightsource.ca (W.C.)

**Keywords:** transition metal boride, hardness, XAFS, thermal stability

## Abstract

The precise determination of atomic position of materials is critical for understanding the relationship between structure and properties, especially for compounds with light elements of boron and single or multiple transition metals. In this work, the single crystal X-ray diffraction is employed to analyze the atomic positions of Co_2_MoB_4_ and Fe_2_MoB_4_ with a Ta_3_B_4_-type structure, and it is found that the lengths of B-B bonds connecting the two zig-zag boron chains are 1.86 Å and 1.87 Å, but previously unreported 1.4 Å. Co and Fe atoms occupy the same crystallographic position in lattice for the doped samples and the valence is close to the metal itself, and Co/Fe K-edge X-ray Absorption Fine Structure(XAFS) spectra of borides with different ratios of Co to Fe are collected to detect the local environment and chemical valence of Co and Fe. Vickers hardness and nano indentation measurements are performed, together with the Density Functional Theory (DFT) calculations. Finally, Co_2_MoB_4_ possess better thermal stability than Fe_2_MoB_4_ evaluated by Thermogravimetric Differential Thermal Analysis (TG-DTA) results.

## 1. Introduction

Transition metal borides possess the interesting physical and chemical properties due to the complex interaction between boron and boron, transition metals and boron in the compounds; thus, the correlation of the structure and properties of borides have been widely investigated in recent years [1,2]. The configuration of boron especially plays an essential role for the borides on the mechanical property, magnetism, superconductivity, catalytic properties [3,4,5]. The presence of unusual short Fe-B bond length and boron dodecahedron make FeB_4_ possess superconducting and hard properties [6]. The different configuration of boron layers leads to large difference of hardness in α-MoB_2_ and β-MoB_2_, and the electrochemical hydrogen evolution efficiency is also varied due to differences in electronic structure induced by the flat boron layer in α-MoB_2_ and the puckered boron layer in β-MoB_2_ [7,8,9]. Moreover, the arrays of boron zig-zag chains also significantly affect the hardness of α-MoB and β-MoB [10]. Thus, the understanding of boron configurations in borides are critical for boron-based materials, either from three-dimensional networks, to two-dimensional layers or to one-dimensional chains.

In past decades, the structure and properties of binary borides with facile synthesis conditions have been studied thoroughly. However, the synthesis of phase-pure ternary borides with a comprehensive structure and constituent elements remain challenging due to their increasing species. Moreover, the different synthesis methods were applied for ternary borides and extensive applications of ternary borides were also explored [11,12,13,14,15]. Importantly, identification of boron atom positions in these ternary borides is a challenge because of the light mass and smaller atomic radius of boron atoms. In our previous work, the phase-pure Co_2_MoB_4_ and Fe_2_MoB_4_ were successful synthesized by a high-pressure technique and were used as a highly efficient oxygen evolution catalyst [16]. However, it is found that there is an unusual short B-B bond connecting double zig-zag boron chains with bond length of 1.4 Å in the lattice provided by previous work [17,18]. Such a short bond length is not common in other borides and boron-containing compounds [19]. This uncertainty remains for more than 50 years. The compounds are also applied as good hydrogen evolution reaction (HER) catalysts and HER property has a close relationship with the structure/electronic structure [16]. Moreover, the understanding of structure can have detailed information of other physical properties, such as superconductivity and mechanical properties.

In this work, we re-determine the atomic positions of Co_2_MoB_4_ and Fe_2_MoB_4_, the crystallographic atomic sites and bond lengths are corrected by the single crystal X-ray diffraction analysis. We also obtain the mechanical properties through the measurements of Vickers hardness and nano indentation, together with DFT calculations. TG-DTA curves of the two compounds and Co, Fe co-doped samples are also measured, the results illustrate that Co_0.5_Fe_1.5_MoB_4_ shows a much better thermal stability. The investigation of structure, mechanical property, chemical valence and thermal stability of the ternary borides indicate that the identification of precise atomic positions for the multi-elements transition metal borides is important to establish the relationship between the structure and physical and chemical properties.

## 2. Materials and Methods

### 2.1. Materials

The raw materials are purchased from Aladdin. The purity of all the materials is 99.9%. The diameters of Co and Fe powder are about 100 nm, and the diameter of Mo powder is about 60 to 200 nm with a specific surface area of 3 to 8 m^2^/g. The diameter of B powder is about 20 μm.

### 2.2. Synthesis Method

The samples were synthesized through a high-temperature and high-pressure (HTHP) technique in a cubic press (GY420 type, Guilin, China). A single substance was weighted in a specific mole ratio (for example, Co:Mo:B = 2:1:4, Fe:Mo:B = 1.9:1:4) and then mixed for more than half an hour in a glovebox to guarantee the homogeneity. The powder was compacted into cylinder shape (about Φ 5 mm × 4 mm) under 10 MPa and then loaded into h-BN capsule surrounded by a graphite heater. The synthesis pressure was set to 5 GPa. The temperature was kept at 1400/1500 °C for half an hour at an increasing rate of 10 °C/s. 

### 2.3. Characterizations

A metallic dark block-shaped single crystal of Fe_2_MoB_4_ (Co_2_MoB_4_, CoFeMoB_4_) with dimensions of 0.015 mm × 0.013 mm × 0.012 mm^3^ (0.016 mm × 0.015 mm × 0.012 mm, 0.018 mm × 0.015 mm × 0.014 mm) was selected and fixed on the top of a thin glass fiber. The single crystal X-ray diffraction data were collected on a Bruker D8 Venture four-circle diffractometer with multilayer monochromator Mo Kα radiation (λ = 0.71073 Å) at 293 K. Data integration and oblique correction was performed with the software package of SAINT. Absorption correction was applied by using the program SADABS. Powder X-ray diffraction (XRD) patterns were obtained using an X-ray diffractometer (PANalytical Empyrean powder X-ray diffractometer) with Cu Kα radiation (λ = 1.54 Å) at a voltage of 40 kV and a current of 40 mA. The VESTA program is used to visualize the crystal structure. The ex-situ Fe K-edge and Co K-edge X-ray Absorption Fine Structure data were collected on the Hard X-ray Micro Analysis (HXMA) beamline at the Canadian Light Source. The Vickers hardness is tested by the Hardness Tester (Qness 60 A+). Elastic modulus measurements were performed using the nanoindenter (Keysight-G200) at room temperature. Thermogravimetric Differential Thermal Analysis are measured by automatic differential thermal balance (HENVEN HQT-4) from 30 °C to 1100 °C at a heating rate of 10 °C/min. The elastic constants (C_ij_) were calculated by the VASP package with the experimental structure of Co_2_MoB_4_ and Fe_2_MoB_4_, the bulk modulus (B), shear modulus (G), Young’s modulus (E) and Poisson’s ratio ν were obtained by the method of Voigt-Reuss-Hill approximation [20,21].

## 3. Results

**Figure 1:**
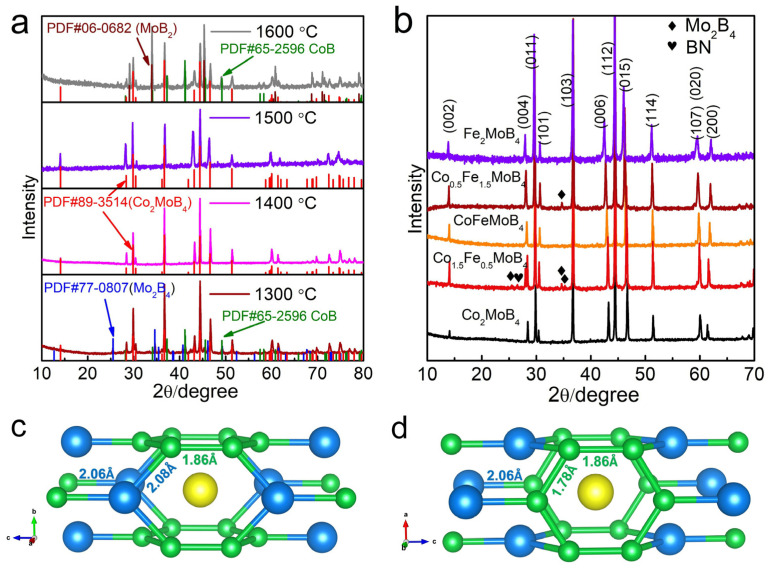
(**a**) XRD patterns of Co_2_MoB_4_ synthesized at 1300 °C, 1400 °C, 1500 °C, 1600 °C; (**b**) XRD patterns of Co_2_MoB_4_, Fe_2_MoB_4_ and Co, Fe co-doped samples; (**c**,**d**) are a scheme of crystal structure of Co_2_MoB_4_ (yellow spheres are Mo atoms, green spheres are B atoms, blue spheres are Co atoms).

Stoichiometric Co_2_MoB_4_ was synthesized by the cubic high-pressure apparatus, a technique that is frequently used for industrial synthetic diamond production [22,23,24,25,26]. We first try to synthesize the boride at different temperatures of 1300 °C, 1400 °C, 1500 °C, 1600 °C in order to screen the suitable synthesis temperature. As shown in Figure 1a, the sample at 1300 °C contains three phases, major Co_2_MoB_4_ and the residual β-MoB_2_ (PDF# 77-0807) and CoB (PDF# 65-2596). As the temperature increases to 1400 °C and 1500 °C, pure Co_2_MoB_4_ is realized in the product. However, two minor phases appear again at the temperature of 1600 °C. From the observed products, we find that the reaction between β-MoB_2_ and CoB at a high temperature promotes the formation of Co_2_MoB_4_. The synthesis of Fe_2_MoB_4_ shows a similar scenario (Figure S1) and the best sintering temperature is around 1500 °C. The results highlight the effect of entropy on the synthesis of phase-pure Co(Fe)_2_MoB_4_ with Ta_3_B_4_-type structure [16]. Then, Co, Fe co-doped samples are also synthesized with ratio of 1:3, 2:2 and 3:1, and the XRD patterns are shown in Figure 1b. The observed change is the lower diffraction angle of (002), (004), (006) lattice planes and higher diffraction angle of (200) lattice plane with the decrease ratio of Co to Fe, suggesting that the decreasing ratio of cobalt to iron induces the expansion of the *a* axis and the compression of the *c* axis.

In order to understand the structure precisely, the single crystal X-ray diffraction analysis was performed in comparison with the previous report listed in Table 1, Table 2 and Tables S1–S15. As we can see, the obtained lattice parameters agree well with the previous work. We take the Co_2_MoB_4_ as an example to illustrate the crystal structure. As shown in Figure 1c,d, the double zig-zag boron chains with equal bond length of 1.78 Å along *b* axis are connected by a longer B-B bond (1.86 Å). The bond lengths between 1.7 Å and 1.89 Å are the typical B-B single bonds, presented either in the boron single chains (MB), the graphene-like/puckered two-dimensional boron layers (MB_2_) or three-dimensional boron networks in CrB_4_/MnB_4_/FeB_4_ [1,6,9,10,19,27,28,29]. It can also be viewed as one-dimensional polyacene-type (distorted graphene-like) chains, suggesting its strong covalent character. Notably, the previously reported short B-B bond is not presented in our single crystal analysis [16,19]. Furthermore, Mo atoms are located at the center of cuboid formed with eight adjacent boron atoms and the Mo-B bond length is 2.34 Å. The cobalt atoms are located in the same plane with double boron chains and a shortest Co-B bond length of 2.06 Å. Moreover, the Co atom also connects the two B atoms to from another two adjacent polyacene-type B chains with bond lengths of 2.08 Å. This kind of interlaced B polyacene-chain, connected by Co atoms, formed three-dimensional networks which may be a critical factor in their mechanical properties. Furthermore, the metal–metal bond distances are 2.68 Å for Co-Co, 2.77 Å for Co-Mo and 3.01 Å for Mo-Mo. The crystal structure of Fe_2_MoB_4_ is the same as Co_2_MoB_4_, and the bond lengths are also similar due to the close atomic radii of Co and Fe.

**Figure 2:**
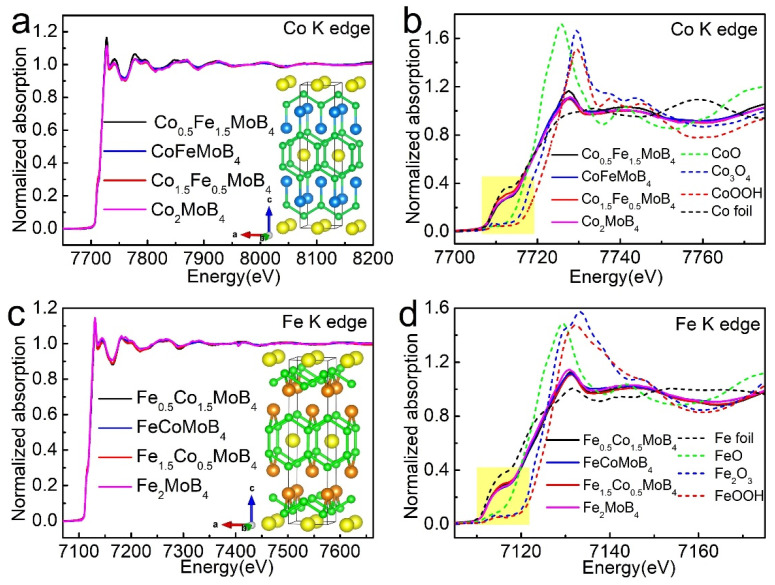
(**a**,**c**) are normalized full XAFS spectra of the Co and Fe K edge of different samples; the insets are structures of Co_2_MoB_4_ and Fe_2_MoB_4_ (Yellow spheres are Mo atoms, green spheres are B atoms, blue spheres are Co atoms and orange spheres are Fe atoms); (**b**,**d**) are normalized XANES of Co and Fe K edge spectra of different samples and some related metal oxides/oxyhydroxides.

To understand the local environment and valence of elements, XAFS is employed to detect the influence of doping for the chemical valence change and coordinate information of Co and Fe [30,31,32,33,34]. The normalized full XAFS spectra of Co and Fe K edges of different samples are illustrated in Figure 2a,c. Throughout the sample system, all the full XAFS spectra of Co/Fe K edge are consistent except for some slight oscillation on intensity despite the diverse content of Co/Fe in the sample, indicating the similarity of the coordinate environment around Co/Fe. Moreover, k^3^ weighted χ(k) spectra of Co, Fe co-doped samples shown in Figure S2 illustrate the high similarity of the Co and Fe local environment in the same sample, suggesting that Co and Fe occupy the same site in the lattice. Fourier transform k^3^ weighted χ(k) spectra are fitted to further confirm the local structure of Co and Fe. The structure models for fitting are listed in Table S16. The fitting results are shown Figures S3 and S4, including magnitude and imaginary part, and the detailed parameters are listed in the Tables S17 and S18. Taking the fitting results of Co_0.5_Fe_1.5_MoB_4_ as an example, all of the Co-centered and Fe-centered shells are close to each other, and the maximum difference is about 0.03 Å, indicating the same coordinate environment of Co and Fe. In addition, the distances and coordinate numbers of some paths, especially for the boron shells, have slight differences in comparison with the structure model calculated by the single crystal X-ray diffraction pattern. The reason for this is that the inevitable formation of the surface oxide layer contributes to the Fourier transformed spectra, specifically, the peaks representing Co/Fe-O paths of oxides between 1 Å and 2 Å shown in Figure S5 influence the distances and coordinate the number of adjacent Co/Fe-B paths and the peaks representing Co/Fe-Co/Fe paths of oxides between 2 Å and 3 Å, which in turn affect the distances and coordinate numbers of Co/Fe-Mo and Co/Fe-Co/Fe paths.

The Co and Fe K edge X-ray Absorption Near Edge Structure (XANES) of synthesized samples associated with related metal and oxides/oxyhydroxides as model compounds are shown in Figure 2b,d due to its sensitivity to the valence. The distinct shape of lines shown in the yellow shadow suggest the symmetry of coordination polyhedron formed by adjacent atoms, and the full absorption edge, including the white line of Co/Fe K edge XANES throughout the sample system and various oxides/oxyhydroxides indicate the intrinsic different valences. Based on the fact that all the spectra of Co/Fe from these compounds are almost identical in spite of different partitioning ratios, it can be concluded that the doping process does not affect the valence of Co/Fe significantly [35], which is different from the doping of sulfides [36]. It is worth noting that the curves of Co from compounds at an energy range of 7708 eV and 7725 eV are closer to the Co foil compared with the scenario of Fe, suggesting that the valence of Fe is slightly higher than Co. Furthermore, the first derivative curves of Co and Fe K absorption edges are also shown in Figure S6 to show the features of absorption edge clearly, and the peak “a” represent transition of electron from 1 s to 3 d orbital. The electron localization function (ELF) shown in Figure S7 also illustrate a weak interaction between Co/Fe and B. The gap between Fe foil and Fe-containing samples at an energy range of 7120 eV and 7130 eV (Figure S6a) suggest that Fe possess a higher valence, which is different to the scenario of Co. Thus, more charge transfers from Fe to adjacent elements due to lower electronegativity of Fe, and the high revolution XPS results of previous work also illustrate this point [16].

**Figure 3:**
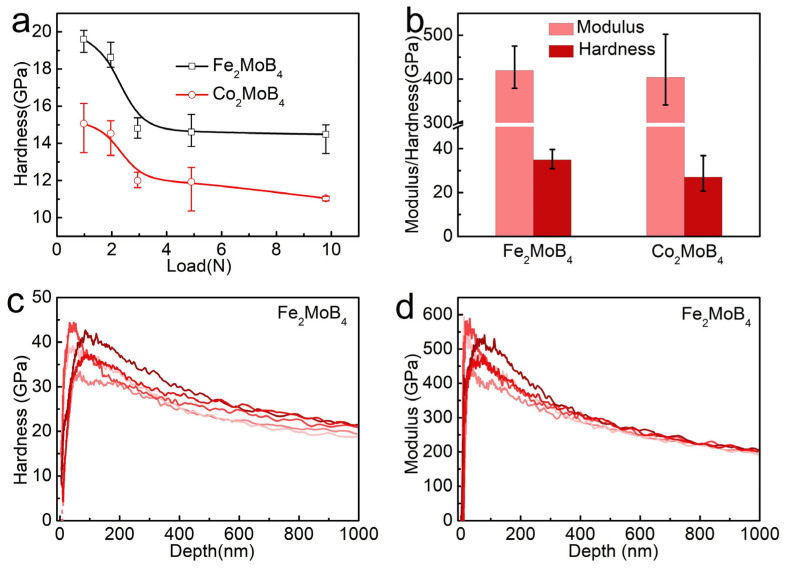
(**a**) Hardness of Fe_2_MoB_4_ and Co_2_MoB_4_ at different loads; (**b**) the average elastic modulus and hardness at depth range of 100 and 200 nm; (**c**,**d**) show the Poisson’s ratio corrected experimental hardness and modulus curves of Fe_2_MoB_4_, respectively.

The mechanical properties, including Vickers’ hardness, elastic moduli of Fe_2_MoB_4_ and Co_2_MoB_4_, are also obtained to explore the relationship between the structure and mechanical property. Figure 3a illustrates the Vickers’ hardness of Fe_2_MoB_4_ and Co_2_MoB_4_ at different loads measured by Vickers’ hardness tester. The Vickers’ hardness of Fe_2_MoB_4_ and Co_2_MoB_4_ are about 19.6 GPa and 15.1 GPa at a load of 0.98 N. The hardness is 14.5 GPa and 11.0 GPa at 9.8 N for Fe_2_MoB_4_ and Co_2_MoB_4_, respectively. The Young’s moduli and hardness of Fe_2_MoB_4_ and Co_2_MoB_4_ at a depth range of 100 and 200 nm are measured by a nano-indentation method as shown in Figure 3b, and the obtained curves are shown in Figure 3c,d, Figures S8a,b and S9. The Young’s moduli of Fe_2_MoB_4_ and Co_2_MoB_4_ are 404 GPa and 420 GPa, approaching the calculated values shown in Table 3, and the measured hardnesses are 27.1 GPa and 34.9 GPa [37]. However, the average hardness of Fe_2_MoB_4_ at a load of 0.25 N and a pressed depth of 1 μm from Figure 3c is 20.4 GPa, and that is obviously different from the average hardness value at a depth range of 100 and 200 nm, indicating the marked influence of load on the hardness of Fe_2_MoB_4_. Moreover, the average hardness of Co_2_MoB_4_ at a load of 0.25 N and a pressed depth of 1 μm from Figures S6a and S7a is 21.5 GPa, which is even harder than that of Fe_2_MoB_4_. The asymptotic hardness of Fe_2_MoB_4_ and Co_2_MoB_4_ is measured by the nano-indentation method approach to the calculated values.

Electron Localization Function (ELF) of Co_2_MoB_4_ and Fe_2_MoB_4_ are also calculated to understand the mechanical properties shown in Figure S7. The electron density is located at the center of B-B bonds from the polyacene-type boron networks, indicating the strong covalent bonds presented in these compounds. The maximum electron density between Mo and B/Mo is about 0.5, suggesting that it makes a weak contribution to the hardness. As we learned from literature, the quasi-3D boron layers can enhance the anti-shearing ability of materials better than the graphene-like boron layers and thus improve the hardness [9]. In Ta_3_B_4_-type borides, Mn_3_B_4_ has a higher measured hardness than MnB_2_ with same structure as α-MoB_2_ [38], and the calculated hardness of Co_2_MoB_4_ is between the α-MoB_2_ (15.2 GPa) and β-MoB_2_ (22 GPa). We thus infer that the polyacene-type boron network is not the only factor for the enhanced hardness. The Co/Fe as the connector of polyacene-type boron networks also play a critical role for the enhanced hardness, which can be identified by the electron density located between the Co/Fe and B atoms shown in Figure S7b,d [39,40,41]. This is so that the Co/Fe-B bonds are connected to the polyacene-type boron networks, which contribute to the hardness of Fe_2_MoB_4_ and Co_2_MoB_4_ predominantly. 

**Figure 4:**
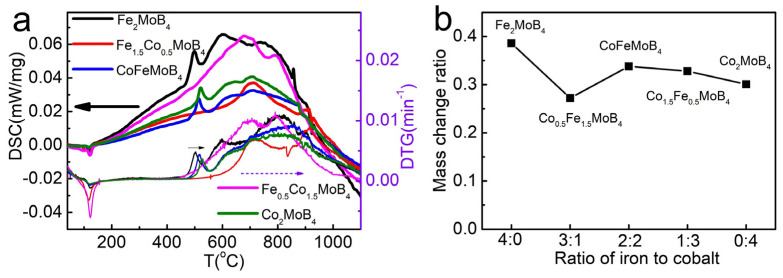
(**a**) Mass normalized DSC and DTG curves of different samples; (**b**) the mass change percentage of different samples after TG-DTA measurement.

The mass normalized Differential Scanning Calorimetry (DSC) and Derivative Thermogravimetric (DTG) curves of samples with different Co/Fe ratios are also given in Figure 4a in order to understand the thermal stability. The obtained TG-DTA and DSC-DTG results are also shown in Figure S10. The exothermic peaks shift from 499 °C to 514 °C and to 521 °C for Fe_2_MoB_4_, CoFeMoB_4_ and Co_2_MoB_4_, and the temperature of peaks representing the fastest mass increase rate are 2 °C to 4 °C higher than that of exothermic peaks. The exothermic peaks of DSC for FeB and CoB are both at around 500 °C [42,43]. Combined with the mass normalized DTG curves, these temperatures represent the start of rapid oxidation, which is also increased with the Co content in the samples. The most interesting phenomenon is that the samples with 25% Fe or Co have no apparent exothermic peaks on the DSC curves and also have no mass increase peaks on the DTG curves at around 500 °C. The most notable sample is Co_0.5_Fe_1.5_MoB_4_, because the slowly increased mass increasing rate start from about 600 °C, indicating a much better thermal stability than the other samples. The mass change ratio after the TG measurements shown in Figure 4b also confirm the unusual thermal stability of Co_0.5_Fe_1.5_MoB_4_.

## 4. Conclusions

In this work, we synthesized the ternary transition metal borides with the Ta_3_B_4_-type structure by a high-pressure and high-temperature method. The precise atomic positions of Fe_2_MoB_4_ and Co_2_MoB_4_ are redetermined by the single crystal X-ray diffraction analysis. The previously unusual short B-B bond is excluded. XAFS of Co_2_MoB_4_ and Fe_2_MoB_4_ are associated with Co and Fe co-doped samples, which illustrate the same position of Co and Fe in the lattice. The valence of Co and Fe are close to the metal itself and does not change with the various doping contents. The experimental hardness and moduli, together with DFT calculations, indicate the hard feature of these borides. The DSC and DTG curves of Co_0.5_Fe_1.5_MoB_4_ present no sharp exothermic peaks and a sudden weight increasing process at around 500 °C, indicating its excellent thermal stability.

## Figures and Tables

**Table 1:** A brief summary of the lattice parameter and atomic parameters of Co_2_MoB_4_ and Fe_2_MoB_4_ in this work and previous reference.

	Co_2_MoB_4_	Co_2_MoB_4_ (Ref. [17])	Fe_2_MoB_4_	Fe_2_MoB_4_ (Ref. [18])
Space group	*Immm*	*Immm*	*Immm*	*Immm*
a (Å)	3.0129	3.079	2.9869	3.128
b (Å)	3.0725	12.57	3.0972	12.7
c (Å)	12.5240	3.018	12.750	2.984
V (Å^3^)	115.94	116.806	117.95	118.541
Atomic parameters (x/a, y/b, z/c)				
Co/Fe	0.5, 0.5, 0.3140	0, 0.18, 0	0.5, 0.5, 0.3151	0, 0.18, 0
B1	1.0, 0, 0.3508	0, 0.375, 0	0.5, 0, 0.4267	0, 0.375, 0
B2	0.5, 0, 0.4259	0, 0.444, 0.5	0, 0, 0.3513	0, 0.444, 0.5
Mo	1.0, 0.5, 0.5	0.5, 0.5, 0	0, 0.5, 0.5	0.5, 0.5, 0

**Table 2:** A brief summary of bond lengths of synthesized compounds determined by X-ray single crystal diffraction.

	Co_2_MoB_4_	Co_2_MoB_4_ (Ref. [17])		Fe_2_MoB_4_	Fe_2_MoB_4_ (Ref. [18])
Bond	Bond length (Å)		Bond	Bond length (Å)	
B-B	1.776 (5)	1.41	B-B	1.776 (11)	1.42
B-B	1.856 (15)	1.74	B-B	1.87 (3)	1.73
Co-B	2.064 (7)	2.19	Fe-B	2.103 (10)	2.22
Co-B	2.2005 (14)	2.45	Fe-B	2.200 (3)	2.48
Co-B	2.080 (5)	2.26	Fe-B	2.122 (15)	2.27
Co-Co	2.6830 (10)	2.78	Fe-Fe	2.718 (2)	2.8
Mo-B	2.343 (3)	2.2	Mo-B	2.346 (6)	2.23
Mo-B	2.419 (5)	2.27	Mo-B	2.448 (11)	2.28

**Table 3:** The calculated bulk modulus, shear modulus, Young’s modulus, Poisson’s ratio and hardness of Co_2_MoB_4_ and Fe_2_MoB_4_.

Sample	Bulk Modulus (GPa)	Shear Modulus (GPa)	Young’s Modulus (GPa)	Poisson’s Ratio	Hardness (GPa)
Co_2_MoB_4_	311.6	181.2	455.3	0.256	19.2
Fe_2_MoB_4_	314.6	178.2	449.6	0.262	18.3

## Data Availability

The Accession CodesCCDC 2157236, 2157237, 2157238 contains the supplementary crystallographic data for this paper. These data can be obtained free of charge via www.ccdc.cam.ac.uk/data_request/cif, or by emailing data_request@ccdc.cam.ac.uk, or by contacting The Cambridge Crystallographic Data Centre, 12 Union Road, Cambridge CB2 1EZ, UK; fax: +44-1223-336033.

## Supplementary Materials

The following supporting information can be downloaded at: https://www.mdpi.com/article/10.3390/ma15093031/s1, Figure S1: XRD pattern of Fe_2_MoB_4_ synthesized at 1400 °C, 1500 °C, 1600 °C; Figure S2: k^3^ weighted χ(k) spectra of Co, Fe co-doped samples, the first element is the absorb element; Figure S3: (a–c) are Fourier transformed k^3^χ(k) oscillations measured at Co K-edge fitted by structure models of Co_2_MoB_4_; (d) Fourier transformed k^3^χ(k) oscillations measured at Co K-edge fitted by structure models of Fe_2_MoB_4_; Figure S4: (a–c) are Fourier transformed k^3^χ(k) oscillations measured at Fe K-edge fitted by structure models of Fe_2_MoB_4_; (d) Fourier transformed k^3^χ(k) oscillations measured at Fe K-edge fitted by structure models of Co_2_MoB_4_; Figure S5: Fourier transformed k^3^ weight χ(k) of Co and Fe related oxides/oxyhydroxides; Figure S6. (a) First derivative curves of different samples at Fe K edge XANES; (b) first derivative curves of different samples at Co K edge XANES; Figure S7. (a,b) The electron localization function (ELF) on the *ac* and *bc* plane for Co_2_MoB_4_. The pink, blue and green spheres represent Mo atoms, Co atoms and B atoms, respectively; (c,d) the electron localization function (ELF) on the *ac* and *bc* plane for Fe_2_MoB_4_. The pink, brown and green spheres represent Mo atoms, Fe atoms and B atoms, respectively. Figure S8: Curves of relationship between hardness/modulus and depth for Co_2_MoB_4_; Figure S9: Curves of relationship between hardness/modulus and load for Co_2_MoB_4_ and Fe_2_MoB_4_; Figure S10. (a) TG-DTA curves of all the samples; (b) DSC-DTG curves of all the sample; Tables S1, S6 and S11: Sample data and structure refinement for Co_2_MoB_4_/Fe_2_MoB_4_/CoFeMoB_4_; Tables S2, S7 and S12: Atomic coordinates and equivalent isotropic atomic displacement parameters (Å^2^) for Co_2_MoB_4_/Fe_2_MoB_4_/CoFeMoB_4_; Tables S3, S8 and S13: Anisotropic atomic displacement parameters (Å^2^) for Co_2_MoB_4_/Fe_2_MoB_4_/CoFeMoB_4_; Tables S4, S9 and S14: Bond lengths (Å) for Co_2_MoB_4_/Fe_2_MoB_4_/CoFeMoB_4_; Tables S5, S10 and S15: Bond angles (°) for Co_2_MoB_4_/Fe_2_MoB_4_/CoFeMoB_4_; Table S16: A brief summary of Paths of Co_2_MoB_4_ and Fe_2_MoB_4_ calculated by Artemis and these parameters are used as model for EXAFS fitting; Table S17: Fitting results parameters of Co K-edge EXAFS of Co_2_MoB_4_ and Fe doped Co_2_MoB_4_; Table S18: Fitting result parameters of Fe K-edge EXAFS of Fe_2_MoB_4_ and Co doped Fe_2_MoB_4_.
