# Supplementary material for: Structure Determination, Mechanical Properties, Thermal Stability of Co2MoB4 and Fe2MoB4
Source: Materials (Basel). 2022 Apr 21;15(9):3031. doi: 10.3390/ma15093031 (PMC9102238; doi:10.3390/ma15093031)
Supplement: Supplementary file 1 [file materials-15-03031-s001.zip › materials-1683738-supplementary.pdf]

Communication

Precise structure determination, mechanical properties, thermal stability of Co_2MoB_4 and Fe_2MoB_4

Shijing Zhao [1], Wenju Zhou [1], Xiaojun Xiang [2], Xuyan Cao [1], Ning Chen[3], Weifeng Chen[3], Xiaohui Yu[2], Bingmin Yan[1,*] and Huiyang Gou [1,*]

[1] Center for High Pressure Science and Technology Advanced Research, Beijing 100094, China; shijing.zhao@hpstar.ac.cn(S.Z.);wenju.zhou@hpstar.ac.cn(W.Z.);xuyan.cao@hpstar.ac.cn(X.C.);

[2] Beijing National Laboratory for Condensed Matter Physics, Institute of Physics, Chinese Academy of Sciences, Beijing 100190, China; yuxh@iphy.ac.cn (X.Y.); xiang_xiaojun@sina.com (X.X.)

[3] Canadian Light Source, Saskatoon, Saskatchewan S7N 2V3, Canada; Weifeng.Chen@lightsource.ca (W.C.); Ning.Chen@lightsource.ca (N.C.)

* Correspondence: bingmin.yan@hpstar.ac.cn (B.Y.); huiyang.gou@hpstar.ac.cn (H.G.)

Table S1. Sample data and structure refinement for Co_2MoB_4.

Empirical formula	Co_2MoB_4
Formula weight	257.04
Temperature	296(2) K
Wavelength	0.71073 Å
Crystal system	Orthorhombic
Space group	*Immm*
Unit cell dimensions	a = 3.0129(3) Å α = 90°<br>b = 3.0725(3) Å β = 90°<br>c = 12.5240(12) Å γ = 90°
Volume	115.94(2) $Å^3$
Z	2
Density (calculated)	7.363 g/cm^3
Absorption coefficient	19.06 mm^{-1}
F(000)	232
Crystal size	0.016 x 0.015 x 0.012 mm^3
θ range for data collection	6.51 to 31.27°
Index ranges	-4<=h<=3, -4<=k<=3, -14<=l<=18
Reflections collected	622
Independent reflections	136 [R(int) = 0.0236]
Coverage of independent reflections	99.3%
Refinement method	Full-matrix least-squares on F^2
Data / restraints / parameters	136 / 0/ 17
Goodness-of-fit	1.154
Final R indices [>2σ(I)]	R_{obs} = 0.0209, wR_{obs} = 0.0416
R indices [all data]	R_{all} = 0.0241, wR_{all} = 0.0426
Largest diff. peak and hole	0.752 and -0.813 e·$Å^{-3}$

$R = \Sigma||F_o|-|F_c|| / \Sigma|F_o|$, $wR = \{\Sigma[w(|F_o|^2 - |F_c|^2)^2] / \Sigma[w(|F_o|^4)]\}^{1/2}$ and calc $w=1/[\sigma^2(Fo^2)+(0.0503P)^2+0.0000P]$ where $P=(Fo^2+2Fc^2)/3$

Table S2. Atomic coordinates and equivalent isotropic atomic displacement parameters ($Å^2$) for Co_2MoB_4.

Label	x	y	z	Occupancy	U_{eq}^*
Mo01	1.0	0.5	0.5	1	0.0052(3)
Co02	0.5	0.5	0.31399(6)	1	0.0057(3)
B1	1.0	0.0	0.3508(5)	1	0.0068(12)
B2	0.5	0.0	0.4259(6)	1	0.0097(12)

*U_{eq} is defined as one third of the trace of the orthogonalized U_{ij} tensor.

Table S3. Anisotropic atomic displacement parameters ($Å^2$) for Co_2MoB_4.

Label	U_{11}	U_{22}	U_{33}	U_{12}	U_{13}	U_{23}
Mo01	0.0045(4)	0.0055(4)	0.0057(4)	0	0	0

Fe02	0.0061(4)	0.0063(5)	0.0047(4)	0	0	0
B1	0.005(3)	0.010(3)	0.005(3)	0	0	0
B2	0.008(5)	0.011(3)	0.010(3)	0	0	0

The anisotropic displacement factor exponent takes the form: $-2\pi^2[h^2a^{*2}U_{11} + \ldots + 2hka^*b^*U_{12}]$.

Table S4. Bond lengths (Å) for Co_2MoB_4.

Label	Distances	Label	Distances
Mo01—B2^{i}	2.343 (3)	B1—B2^{vii}	1.776 (5)
Mo01—B2^{ii}	2.343 (3)	B1—Co02^{viii}	2.064 (7)
Mo01—B2^{iii}	2.343 (3)	B1—Co02	2.2005 (14)
Mo01—B2^{iv}	2.343 (3)	B1—Co02^{ix}	2.2005 (14)
Mo01—B2^{v}	2.343 (3)	B1—Co02^{vii}	2.2005 (14)
Mo01—B2^{vi}	2.343 (3)	B1—Co02^{x}	2.2005 (14)
Mo01—B2^{vii}	2.343 (3)	Co02—B2	2.080 (5)
Mo01—B2	2.343 (3)	Co02—B2^{vi}	2.080 (5)
Mo01—B1	2.419 (5)	Co02—Co02^{xi}	2.6830 (10)
Mo01—B1^{i}	2.419 (5)	Co02—Co02^{xii}	2.6830 (9)
Mo01—B1^{v}	2.419 (5)	Co02—Co02^{viii}	2.6830 (10)
Mo01—B1^{vi}	2.419 (5)	Co02—Co02^{xiii}	2.6830 (10)
B1—B2	1.776 (5)	B2—B2^{ii}	1.856 (15)

Symmetry codes: (i) −x+2, −y+1, −z+1; (ii) −x+1, −y, −z+1; (iii) x+1, y+1, z; (iv) −x+1, −y+1, −z+1; (v) −x+2, −y, −z+1; (vi) x, y+1, z; (vii) x+1, y, z; (viii) −x+3/2, −y+1/2, −z+1/2; (ix) x, y−1, z; (x) x+1, y−1, z; (xi) −x+1/2, −y+1/2, −z+1/2; (xii) −x+1/2, −y+3/2, −z+1/2; (xiii) −x+3/2, −y+3/2, −z+1/2; (xiv) x−1, y, z; (xv) x−1, y+1, z; (xvi) x−1, y−1, z.

Table S5. Bond angles (°) for Co_2MoB_4.

Label	Angles	Label	Angles
B2^{i}—Mo01—B2^{ii}	133.3 (3)	B1^{viii}—Co02—B2	132.38 (15)
B2^{i}—Mo01—B2^{iii}	46.7 (3)	B1^{viii}—Co02—B2^{vi}	132.38 (15)
B2^{ii}—Mo01—B2^{iii}	180.0	B2—Co02—B2^{vi}	95.2 (3)
B2^{i}—Mo01—B2^{iv}	80.02 (12)	B1^{viii}—Co02—B1^{vi}	102.10 (17)
B2^{ii}—Mo01—B2^{iv}	81.94 (13)	B2—Co02—B1^{vi}	111.99 (18)
B2^{iii}—Mo01—B2^{iv}	98.06 (13)	B2^{vi}—Co02—B1^{vi}	48.93 (14)
B2^{i}—Mo01—B2^{v}	81.94 (13)	B1^{viii}—Co02—B1^{xiv}	102.10 (17)
B2^{ii}—Mo01—B2^{v}	80.02 (12)	B2—Co02—B1^{xiv}	48.93 (14)
B2^{iii}—Mo01—B2^{v}	99.98 (12)	B2^{vi}—Co02—B1^{xiv}	111.99 (18)
B2^{iv}—Mo01—B2^{v}	133.3 (3)	B1^{vi}—Co02—B1^{xiv}	155.8 (3)
B2^{i}—Mo01—B2^{vi}	98.06 (13)	B1^{viii}—Co02—B1^{xv}	102.10 (17)
B2^{ii}—Mo01—B2^{vi}	99.98 (12)	B2—Co02—B1^{xv}	111.99 (18)
B2^{iii}—Mo01—B2^{vi}	80.02 (12)	B2^{vi}—Co02—B1^{xv}	48.93 (14)
B2^{iv}—Mo01—B2^{vi}	46.7 (3)	B1^{vi}—Co02—B1^{xv}	86.41 (7)

B2^{v}—Mo01—B2^{vi}	180.0 (3)	B1^{xiv}—Co02—B1^{xv}	88.56 (7)
B2^{i}—Mo01—B2^{vii}	99.98 (12)	B1^{viii}—Co02—B1	102.10 (17)
B2^{ii}—Mo01—B2^{vii}	98.06 (13)	B2—Co02—B1	48.93 (14)
B2^{iii}—Mo01—B2^{vii}	81.94 (13)	B2^{vi}—Co02—B1	111.99 (18)
B2^{iv}—Mo01—B2^{vii}	180.0 (3)	B1^{vi}—Co02—B1	88.56 (7)
B2^{v}—Mo01—B2^{vii}	46.7 (3)	B1^{xiv}—Co02—B1	86.41 (7)
B2^{vi}—Mo01—B2^{vii}	133.3 (3)	B1^{xv}—Co02—B1	155.8 (3)
B2^{i}—Mo01—B2	180.0	B1^{viii}—Co02—Co02^{xi}	53.32 (3)
B2^{ii}—Mo01—B2	46.7 (3)	B2—Co02—Co02^{xi}	88.84 (13)
B2^{iii}—Mo01—B2	133.3 (3)	B2^{vi}—Co02—Co02^{xi}	145.653 (18)
B2^{iv}—Mo01—B2	99.98 (12)	B1^{vi}—Co02—Co02^{xi}	155.41 (18)
B2^{v}—Mo01—B2	98.06 (13)	B1^{xiv}—Co02—Co02^{xi}	48.78 (17)
B2^{vi}—Mo01—B2	81.94 (13)	B1^{xv}—Co02—Co02^{xi}	98.08 (10)
B2^{vii}—Mo01—B2	80.02 (12)	B1—Co02—Co02^{xi}	96.31 (10)
B2^{i}—Mo01—B1	136.24 (14)	B1^{viii}—Co02—Co02^{xii}	53.32 (3)
B2^{ii}—Mo01—B1	83.65 (17)	B2—Co02—Co02^{xii}	145.653 (19)
B2^{iii}—Mo01—B1	96.35 (17)	B2^{vi}—Co02—Co02^{xii}	88.84 (13)
B2^{iv}—Mo01—B1	136.24 (14)	B1^{vi}—Co02—Co02^{xii}	96.31 (10)
B2^{v}—Mo01—B1	83.65 (17)	B1^{xiv}—Co02—Co02^{xii}	98.08 (10)
B2^{vi}—Mo01—B1	96.35 (17)	B1^{xv}—Co02—Co02^{xii}	48.78 (17)
B2^{vii}—Mo01—B1	43.76 (14)	B1—Co02—Co02^{xii}	155.42 (18)
B2—Mo01—B1	43.76 (14)	Co02^{xi}—Co02—Co02^{xii}	69.86 (3)
B2^{i}—Mo01—B1^{i}	43.76 (14)	B1^{viii}—Co02—Co02^{viii}	53.32 (3)
B2^{ii}—Mo01—B1^{i}	96.35 (17)	B2—Co02—Co02^{viii}	88.84 (13)
B2^{iii}—Mo01—B1^{i}	83.65 (17)	B2^{vi}—Co02—Co02^{viii}	145.653 (19)
B2^{iv}—Mo01—B1^{i}	43.76 (14)	B1^{vi}—Co02—Co02^{viii}	98.08 (10)
B2^{v}—Mo01—B1^{i}	96.35 (17)	B1^{xiv}—Co02—Co02^{viii}	96.31 (10)
B2^{vi}—Mo01—B1^{i}	83.65 (17)	B1^{xv}—Co02—Co02^{viii}	155.41 (18)
B2^{vii}—Mo01—B1^{i}	136.24 (14)	B1—Co02—Co02^{viii}	48.78 (17)
B2—Mo01—B1^{i}	136.24 (14)	Co02^{xi}—Co02—Co02^{viii}	68.32 (3)
B1—Mo01—B1^{i}	180.0	Co02^{xii}—Co02—Co02^{viii}	106.63 (5)
B2^{i}—Mo01—B1^{v}	96.35 (17)	B1^{viii}—Co02—Co02^{xiii}	53.32 (3)
B2^{ii}—Mo01—B1^{v}	43.76 (14)	B2—Co02—Co02^{xiii}	145.653 (19)
B2^{iii}—Mo01—B1^{v}	136.24 (14)	B2^{vi}—Co02—Co02^{xiii}	88.84 (13)
B2^{iv}—Mo01—B1^{v}	96.35 (17)	B1^{vi}—Co02—Co02^{xiii}	48.78 (17)
B2^{v}—Mo01—B1^{v}	43.76 (14)	B1^{xiv}—Co02—Co02^{xiii}	155.41 (18)
B2^{vi}—Mo01—B1^{v}	136.24 (14)	B1^{xv}—Co02—Co02^{xiii}	96.31 (10)
B2^{vii}—Mo01—B1^{v}	83.65 (17)	B1—Co02—Co02^{xiii}	98.08 (10)
B2—Mo01—B1^{v}	83.65 (17)	Co02^{xi}—Co02—Co02^{xiii}	106.63 (5)
B1—Mo01—B1^{v}	101.1 (2)	Co02^{xii}—Co02—Co02^{xiii}	68.32 (3)

B1^{i}—Mo01—B1^{v}	78.9 (2)	Co02^{viii}—Co02—Co02^{xiii}	69.86 (3)
B2^{i}—Mo01—B1^{vi}	83.65 (17)	B1^{viii}—Co02—Mo01	147.111 (10)
B2^{ii}—Mo01—B1^{vi}	136.24 (14)	B2—Co02—Mo01	55.53 (12)
B2^{iii}—Mo01—B1^{vi}	43.76 (14)	B2^{vi}—Co02—Mo01	55.53 (12)
B2^{iv}—Mo01—B1^{vi}	83.65 (17)	B1^{vi}—Co02—Mo01	56.79 (15)
B2^{v}—Mo01—B1^{vi}	136.24 (14)	B1^{xiv}—Co02—Mo01	101.29 (16)
B2^{vi}—Mo01—B1^{vi}	43.76 (14)	B1^{xv}—Co02—Mo01	101.29 (16)
B2^{vii}—Mo01—B1^{vi}	96.35 (17)	B1—Co02—Mo01	56.79 (15)
B2—Mo01—B1^{vi}	96.35 (17)	Co02^{xi}—Co02—Mo01	143.76 (2)
B1—Mo01—B1^{vi}	78.9 (2)	Co02^{xii}—Co02—Mo01	143.76 (2)
B1^{i}—Mo01—B1^{vi}	101.1 (2)	Co02^{viii}—Co02—Mo01	101.347 (19)
B1^{v}—Mo01—B1^{vi}	180.0	Co02^{xiii}—Co02—Mo01	101.347 (19)
B2—B1—B2^{vii}	116.0 (6)	B1—B2—B1^{xiv}	116.0 (6)
B2—B1—Co02^{viii}	122.0 (3)	B1—B2—B2^{ii}	122.0 (3)
B2^{vii}—B1—Co02^{viii}	122.0 (3)	B1^{xiv}—B2—B2^{ii}	122.0 (3)
B2—B1—Co02	61.99 (13)	B1—B2—Co02	69.1 (2)
B2^{vii}—B1—Co02	133.77 (15)	B1^{xiv}—B2—Co02	69.1 (2)
Co02^{viii}—B1—Co02	77.90 (17)	B2^{ii}—B2—Co02	132.38 (15)
B2—B1—Co02^{ix}	61.99 (13)	B1—B2—Co02^{ix}	69.1 (2)
B2^{vii}—B1—Co02^{ix}	133.77 (15)	B1^{xiv}—B2—Co02^{ix}	69.1 (2)
Co02^{viii}—B1—Co02^{ix}	77.90 (17)	B2^{ii}—B2—Co02^{ix}	132.38 (15)
Co02—B1—Co02^{ix}	88.56 (7)	Co02—B2—Co02^{ix}	95.2 (3)
B2—B1—Co02^{vii}	133.77 (15)	B1—B2—Mo01	70.39 (13)
B2^{vii}—B1—Co02^{vii}	61.99 (13)	B1^{xiv}—B2—Mo01	139.03 (7)
Co02^{viii}—B1—Co02^{vii}	77.90 (17)	B2^{ii}—B2—Mo01	66.67 (17)
Co02—B1—Co02^{vii}	86.41 (7)	Co02—B2—Mo01	77.44 (3)
Co02^{ix}—B1—Co02^{vii}	155.8 (3)	Co02^{ix}—B2—Mo01	138.70 (14)
B2—B1—Co02^{x}	133.77 (15)	B1—B2—Mo01^{xvi}	139.03 (7)
B2^{vii}—B1—Co02^{x}	61.99 (13)	B1^{xiv}—B2—Mo01^{xvi}	70.39 (13)
Co02^{viii}—B1—Co02^{x}	77.90 (17)	B2^{ii}—B2—Mo01^{xvi}	66.67 (17)
Co02—B1—Co02^{x}	155.8 (3)	Co02—B2—Mo01^{xvi}	138.70 (14)
Co02^{ix}—B1—Co02^{x}	86.41 (7)	Co02^{ix}—B2—Mo01^{xvi}	77.44 (3)
Co02^{vii}—B1—Co02^{x}	88.56 (7)	Mo01—B2—Mo01^{xvi}	133.3 (3)
B2—B1—Mo01	65.9 (2)	B1—B2—Mo01^{xiv}	139.03 (7)
B2^{vii}—B1—Mo01	65.9 (2)	B1^{xiv}—B2—Mo01^{xiv}	70.39 (13)
Co02^{viii}—B1—Mo01	140.57 (10)	B2^{ii}—B2—Mo01^{xiv}	66.67 (17)
Co02—B1—Mo01	73.65 (8)	Co02—B2—Mo01^{xiv}	77.44 (3)
Co02^{ix}—B1—Mo01	127.25 (19)	Co02^{ix}—B2—Mo01^{xiv}	138.70 (14)
Co02^{vii}—B1—Mo01	73.65 (8)	Mo01—B2—Mo01^{xiv}	80.02 (12)
Co02^{x}—B1—Mo01	127.25 (19)	Mo01^{xvi}—B2—Mo01^{xiv}	81.94 (13)

B2—B1—Mo01^{ix}	65.9 (2)	B1—B2—Mo01^{ix}	70.39 (13)
B2^{vii}—B1—Mo01^{ix}	65.9 (2)	B1^{xiv}—B2—Mo01^{ix}	139.03 (7)
Co02^{viii}—B1—Mo01^{ix}	140.57 (10)	B2^{ii}—B2—Mo01^{ix}	66.67 (17)
Co02—B1—Mo01^{ix}	127.25 (19)	Co02—B2—Mo01^{ix}	138.70 (14)
Co02^{ix}—B1—Mo01^{ix}	73.65 (8)	Co02^{ix}—B2—Mo01^{ix}	77.44 (3)
Co02^{vii}—B1—Mo01^{ix}	127.25 (19)	Mo01—B2—Mo01^{ix}	81.94 (13)
Co02^{x}—B1—Mo01^{ix}	73.65 (8)	Mo01^{xvi}—B2—Mo01^{ix}	80.02 (12)
Mo01—B1—Mo01^{ix}	78.9 (2)	Mo01^{xiv}—B2—Mo01^{ix}	133.3 (3)

Symmetry codes: (i) −x+2, −y+1, −z+1; (ii) −x+1, −y, −z+1; (iii) x+1, y+1, z; (iv) −x+1, −y+1, −z+1; (v) −x+2, −y, −z+1; (vi) x, y+1, z; (vii) x+1, y, z; (viii) −x+3/2, −y+1/2, −z+1/2; (ix) x, y−1, z; (x) x+1, y−1, z; (xi) −x+1/2, −y+1/2, −z+1/2; (xii) −x+1/2, −y+3/2, −z+1/2; (xiii) −x+3/2, −y+3/2, −z+1/2; (xiv) x−1, y, z; (xv) x−1, y+1, z; (xvi) x−1, y−1, z.

Table S6. Sample data and structure refinement for Fe_2MoB_4

Empirical formula	Fe_2MoB_4
Formula weight	250.88
Temperature	296(2) K
Wavelength	0.71073 Å
Crystal system	orthorhombic
Space group	*I*mmm
Unit cell dimensions	a = 2.9869(5) Å α = 90°<br>b = 3.0972(5) Å β = 90°<br>c = 12.750(2) Å γ = 90°
Volume	117.95(3) Å^3
Z	2
Density (calculated)	7.064 g/cm^3
Absorption coefficient	16.967 mm^{-1}
F(000)	228
Crystal size	0.012 x 0.013 x 0.015 mm^3
θ range for data collection	3.19 to 30.36°
Index ranges	-3<=h<=4, -4<=k<=4, -16<=l<=18
Reflections collected	502
Independent reflections	129 [R(int) = 0.0378]
Coverage of independent reflections	100%
Refinement method	Full-matrix least-squares on F^2
Data / restraints / parameters	129 / 12 / 16
Goodness-of-fit	1.236
Final R indices [>2σ(I)]	R_{obs} = 0.0442, wR_{obs} = 0.0732
R indices [all data]	R_{all} = 0.0566, wR_{all} = 0.0784
Largest diff. peak and hole	1.948 and -1.922 e·Å^{-3}

$R = \Sigma||F_o|-|F_c|| / \Sigma|F_o|$, $wR = \{\Sigma[w(|F_o|^2 - |F_c|^2)^2] / \Sigma[w(|F_o|^4)]\}^{1/2}$ and calc $w=1/[\sigma^2(Fo^2)+(0.0503P)^2+0.0000P]$ where $P=(Fo^2+2Fc^2)/3$

Table S7. Atomic coordinates and equivalent isotropic atomic displacement parameters (Å^2) for Fe_2MoB_4.

Label	x	y	z	Occupancy	U_{eq}*
Mo01	0.0	0.5	0.5	1	0.0043(4)
Fe02	0.5	0.5	0.31513(15)	1	0.0052(5)
B1	0.5	0.0	0.4267(11)	1	0.002(3)
B2	0.0	0.0	0.3513(12)	1	0.005(3)

*U_{eq} is defined as one third of the trace of the orthogonalized U_{ij} tensor.

Table S8. Anisotropic atomic displacement parameters (Å^2) for Fe_2MoB_4.

Label	U_{11}	U_{22}	U_{33}	U_{12}	U_{13}	U_{23}
Mo01	0.0040(8)	0.0043(9)	0.0046(8)	0	0	0
Fe02	0.0068(11)	0.0052(11)	0.0036(9)	0	0	0
B1	0.002(5)	0.001(6)	0.002(5)	0	0	0
B2	0.008(5)	0.002(5)	0.004(4)	0	0	0

The anisotropic displacement factor exponent takes the form: $-2\pi^2[h^2a^{*2}U_{11} + ... + 2hka^*b^*U_{12}]$.

Table S9. Bond lengths (Å) for Fe_2MoB_4.

Label	Distances	Label	Distances
Mo01—B1^{i}	2.346 (6)	Fe02—B1	2.103 (10)
Mo01—B1	2.346 (6)	Fe02—B2^{viii}	2.122 (15)
Mo01—B1^{ii}	2.346 (6)	Fe02—B2^{vi}	2.200 (3)
Mo01—B1^{iii}	2.346 (6)	Fe02—B2^{ix}	2.200 (3)
Mo01—B1^{iv}	2.346 (6)	Fe02—B2^{x}	2.200 (3)
Mo01—B1^{v}	2.346 (6)	Fe02—B2	2.200 (3)
Mo01—B1^{vi}	2.346 (6)	Fe02—Fe02^{xi}	2.718 (2)
Mo01—B1^{vii}	2.346 (6)	Fe02—Fe02^{xii}	2.718 (2)
Mo01—B2	2.448 (11)	Fe02—Fe02^{viii}	2.718 (2)
Mo01—B2^{i}	2.448 (11)	Fe02—Fe02^{xiii}	2.718 (2)
Mo01—B2^{v}	2.448 (11)	B1—B2	1.776 (11)
Mo01—B2^{vi}	2.448 (11)	B1—B2^{ix}	1.776 (11)
Fe02—B1^{vi}	2.103 (10)	B1—B1^{ii}	1.87 (3)

Symmetry codes: (i) −x+2, −y+1, −z+1; (ii) −x+1, −y, −z+1; (iii) x+1, y+1, z; (iv) −x+1, −y+1, −z+1; (v) −x+2, −y, −z+1; (vi) x, y+1, z; (vii) x+1, y, z; (viii) −x+3/2, −y+1/2, −z+1/2; (ix) x−1, y, z; (x) x−1, y+1, z; (xi) −x+1/2, −y+1/2, −z+1/2; (xii) −x+1/2, −y+3/2, −z+1/2; (xiii) −x+3/2, −y+3/2, −z+1/2; (xiv) x, y−1, z; (xv) x−1, y−1, z; (xvi) x+1, y−1, z.

Table S10. Bond angles (°) for Fe_2MoB_4.

Label	Angles	Label	Angles
B1^{i}—Mo01—B1	180.0000	B1^{vi}—Fe02—Fe02^{viii}	146.41 (4)
B1^{i}—Mo01—B1^{ii}	133.0 (6)	B1—Fe02—Fe02^{viii}	89.6 (2)
B1—Mo01—B1^{ii}	47.0 (6)	B2^{viii}—Fe02—Fe02^{viii}	52.33 (6)

B1^{i}—Mo01—B1^{iii}	47.0 (6)	B2^{vi}—Fe02—Fe02^{viii}	99.0 (2)
B1—Mo01—B1^{iii}	133.0 (6)	B2^{ix}—Fe02—Fe02^{viii}	95.7 (2)
B1^{ii}—Mo01—B1^{iii}	180.0000	B2^{x}—Fe02—Fe02^{viii}	154.4 (4)
B1^{i}—Mo01—B1^{iv}	79.1 (2)	B2—Fe02—Fe02^{viii}	49.8 (4)
B1—Mo01—B1^{iv}	100.9 (2)	Fe02^{xi}—Fe02—Fe02^{viii}	66.66 (6)
B1^{ii}—Mo01—B1^{iv}	82.6 (2)	Fe02^{xii}—Fe02—Fe02^{viii}	104.66 (12)
B1^{iii}—Mo01—B1^{iv}	97.4 (2)	B1^{vi}—Fe02—Fe02^{xiii}	89.6 (2)
B1^{i}—Mo01—B1^{v}	82.6 (2)	B1—Fe02—Fe02^{xiii}	146.41 (4)
B1—Mo01—B1^{v}	97.4 (2)	B2^{viii}—Fe02—Fe02^{xiii}	52.33 (6)
B1^{ii}—Mo01—B1^{v}	79.1 (2)	B2^{vi}—Fe02—Fe02^{xiii}	49.8 (4)
B1^{iii}—Mo01—B1^{v}	100.9 (2)	B2^{ix}—Fe02—Fe02^{xiii}	154.4 (4)
B1^{iv}—Mo01—B1^{v}	133.0 (6)	B2^{x}—Fe02—Fe02^{xiii}	95.7 (2)
B1^{i}—Mo01—B1^{vi}	97.4 (2)	B2—Fe02—Fe02^{xiii}	99.0 (2)
B1—Mo01—B1^{vi}	82.6 (2)	Fe02^{xi}—Fe02—Fe02^{xiii}	104.66 (12)
B1^{ii}—Mo01—B1^{vi}	100.9 (2)	Fe02^{xii}—Fe02—Fe02^{xiii}	66.66 (6)
B1^{iii}—Mo01—B1^{vi}	79.1 (2)	Fe02^{viii}—Fe02—Fe02^{xiii}	69.47 (7)
B1^{iv}—Mo01—B1^{vi}	47.0 (6)	B1^{vi}—Fe02—Mo01	55.2 (2)
B1^{v}—Mo01—B1^{vi}	180.0 (6)	B1—Fe02—Mo01	55.2 (2)
B1^{i}—Mo01—B1^{vii}	100.9 (2)	B2^{viii}—Fe02—Mo01	147.64 (2)
B1—Mo01—B1^{vii}	79.1 (2)	B2^{vi}—Fe02—Mo01	57.3 (3)
B1^{ii}—Mo01—B1^{vii}	97.4 (2)	B2^{ix}—Fe02—Mo01	100.7 (4)
B1^{iii}—Mo01—B1^{vii}	82.6 (2)	B2^{x}—Fe02—Mo01	100.7 (4)
B1^{iv}—Mo01—B1^{vii}	180.0 (6)	B2—Fe02—Mo01	57.3 (3)
B1^{v}—Mo01—B1^{vii}	47.0 (6)	Fe02^{xi}—Fe02—Mo01	144.12 (5)
B1^{vi}—Mo01—B1^{vii}	133.0 (6)	Fe02^{xii}—Fe02—Mo01	144.12 (5)
B1^{i}—Mo01—B2	136.6 (3)	Fe02^{viii}—Fe02—Mo01	102.83 (4)
B1—Mo01—B2	43.4 (3)	Fe02^{xiii}—Fe02—Mo01	102.83 (4)
B1^{ii}—Mo01—B2	83.7 (3)	B2—B1—B2^{ix}	114.5 (11)
B1^{iii}—Mo01—B2	96.3 (3)	B2—B1—B1^{ii}	122.8 (6)
B1^{iv}—Mo01—B2	136.6 (3)	B2^{ix}—B1—B1^{ii}	122.8 (6)
B1^{v}—Mo01—B2	83.7 (3)	B2—B1—Fe02	68.5 (4)
B1^{vi}—Mo01—B2	96.3 (3)	B2^{ix}—B1—Fe02	68.5 (4)
B1^{vii}—Mo01—B2	43.4 (3)	B1^{ii}—B1—Fe02	132.6 (3)
B1^{i}—Mo01—B2^{i}	43.4 (3)	B2—B1—Fe02^{xiv}	68.5 (4)
B1—Mo01—B2^{i}	136.6 (3)	B2^{ix}—B1—Fe02^{xiv}	68.5 (4)
B1^{ii}—Mo01—B2^{i}	96.3 (3)	B1^{ii}—B1—Fe02^{xiv}	132.6 (3)
B1^{iii}—Mo01—B2^{i}	83.7 (3)	Fe02—B1—Fe02^{xiv}	94.9 (6)
B1^{iv}—Mo01—B2^{i}	43.4 (3)	B2—B1—Mo01	71.4 (3)
B1^{v}—Mo01—B2^{i}	96.3 (3)	B2^{ix}—B1—Mo01	138.68 (13)
B1^{vi}—Mo01—B2^{i}	83.7 (3)	B1^{ii}—B1—Mo01	66.5 (3)

$B1^{vii}$—Mo01—$B2^{i}$	136.6 (3)	Fe02—B1—Mo01	77.49 (6)
B2—Mo01—$B2^{i}$	180.0 (4)	$Fe02^{xiv}$—B1—Mo01	139.1 (3)
$B1^{i}$—Mo01—$B2^{v}$	96.3 (3)	B2—B1—$Mo01^{xv}$	138.68 (13)
B1—Mo01—$B2^{v}$	83.7 (3)	$B2^{ix}$—B1—$Mo01^{xv}$	71.4 (3)
$B1^{ii}$—Mo01—$B2^{v}$	43.4 (3)	$B1^{ii}$—B1—$Mo01^{xv}$	66.5 (3)
$B1^{iii}$—Mo01—$B2^{v}$	136.6 (3)	Fe02—B1—$Mo01^{xv}$	139.1 (3)
$B1^{iv}$—Mo01—$B2^{v}$	96.3 (3)	$Fe02^{xiv}$—B1—$Mo01^{xv}$	77.49 (6)
$B1^{v}$—Mo01—$B2^{v}$	43.4 (3)	Mo01—B1—$Mo01^{xv}$	133.0 (6)
$B1^{vi}$—Mo01—$B2^{v}$	136.6 (3)	B2—B1—$Mo01^{xiv}$	71.4 (3)
$B1^{vii}$—Mo01—$B2^{v}$	83.7 (3)	$B2^{ix}$—B1—$Mo01^{xiv}$	138.68 (13)
B2—Mo01—$B2^{v}$	101.5 (4)	$B1^{ii}$—B1—$Mo01^{xiv}$	66.5 (3)
$B2^{i}$—Mo01—$B2^{v}$	78.5 (4)	Fe02—B1—$Mo01^{xiv}$	139.1 (3)
$B1^{i}$—Mo01—$B2^{vi}$	83.7 (3)	$Fe02^{xiv}$—B1—$Mo01^{xiv}$	77.49 (6)
B1—Mo01—$B2^{vi}$	96.3 (3)	Mo01—B1—$Mo01^{xiv}$	82.6 (2)
$B1^{ii}$—Mo01—$B2^{vi}$	136.6 (3)	$Mo01^{xv}$—B1—$Mo01^{xiv}$	79.1 (2)
$B1^{iii}$—Mo01—$B2^{vi}$	43.4 (3)	B2—B1—$Mo01^{ix}$	138.68 (13)
$B1^{iv}$—Mo01—$B2^{vi}$	83.7 (3)	$B2^{ix}$—B1—$Mo01^{ix}$	71.4 (3)
$B1^{v}$—Mo01—$B2^{vi}$	136.6 (3)	$B1^{ii}$—B1—$Mo01^{ix}$	66.5 (3)
$B1^{vi}$—Mo01—$B2^{vi}$	43.4 (3)	Fe02—B1—$Mo01^{ix}$	77.49 (6)
$B1^{vii}$—Mo01—$B2^{vi}$	96.3 (3)	$Fe02^{xiv}$—B1—$Mo01^{ix}$	139.1 (3)
B2—Mo01—$B2^{vi}$	78.5 (4)	Mo01—B1—$Mo01^{ix}$	79.1 (2)
$B2^{i}$—Mo01—$B2^{vi}$	101.5 (4)	$Mo01^{xv}$—B1—$Mo01^{ix}$	82.6 (2)
$B2^{v}$—Mo01—$B2^{vi}$	180.0000	$Mo01^{xiv}$—B1—$Mo01^{ix}$	133.0 (6)
$B1^{vi}$—Fe02—B1	94.9 (6)	$B1^{vii}$—B2—B1	114.5 (11)
$B1^{vi}$—Fe02—$B2^{viii}$	132.6 (3)	$B1^{vii}$—B2—$Fe02^{viii}$	122.8 (6)
B1—Fe02—$B2^{viii}$	132.6 (3)	B1—B2—$Fe02^{viii}$	122.8 (6)
$B1^{vi}$—Fe02—$B2^{vi}$	48.7 (3)	$B1^{vii}$—B2—Fe02	133.2 (3)
B1—Fe02—$B2^{vi}$	112.1 (4)	B1—B2—Fe02	62.8 (2)
$B2^{viii}$—Fe02—$B2^{vi}$	102.1 (4)	$Fe02^{viii}$—B2—Fe02	77.9 (4)
$B1^{vi}$—Fe02—$B2^{ix}$	112.1 (4)	$B1^{vii}$—B2—$Fe02^{xiv}$	133.2 (3)
B1—Fe02—$B2^{ix}$	48.7 (3)	B1—B2—$Fe02^{xiv}$	62.8 (2)
$B2^{viii}$—Fe02—$B2^{ix}$	102.1 (4)	$Fe02^{viii}$—B2—$Fe02^{xiv}$	77.9 (4)
$B2^{vi}$—Fe02—$B2^{ix}$	155.8 (8)	Fe02—B2—$Fe02^{xiv}$	89.47 (16)
$B1^{vi}$—Fe02—$B2^{x}$	48.7 (3)	$B1^{vii}$—B2—$Fe02^{vii}$	62.8 (2)
B1—Fe02—$B2^{x}$	112.1 (4)	B1—B2—$Fe02^{vii}$	133.2 (3)
$B2^{viii}$—Fe02—$B2^{x}$	102.1 (4)	$Fe02^{viii}$—B2—$Fe02^{vii}$	77.9 (4)
$B2^{vi}$—Fe02—$B2^{x}$	85.50 (15)	Fe02—B2—$Fe02^{vii}$	85.50 (15)
$B2^{ix}$—Fe02—$B2^{x}$	89.47 (16)	$Fe02^{xiv}$—B2—$Fe02^{vii}$	155.8 (8)
$B1^{vi}$—Fe02—B2	112.1 (4)	$B1^{vii}$—B2—$Fe02^{xvi}$	62.8 (2)
B1—Fe02—B2	48.7 (3)	B1—B2—$Fe02^{xvi}$	133.2 (3)

$B2^{viii}$—Fe02—B2	102.1 (4)	$Fe02^{viii}$—B2—$Fe02^{xvi}$	77.9 (4)
$B2^{vi}$—Fe02—B2	89.47 (16)	Fe02—B2—$Fe02^{xvi}$	155.8 (8)
$B2^{ix}$—Fe02—B2	85.50 (15)	$Fe02^{xiv}$—B2—$Fe02^{xvi}$	85.50 (15)
$B2^{x}$—Fe02—B2	155.8 (8)	$Fe02^{vii}$—B2—$Fe02^{xvi}$	89.47 (16)
$B1^{vi}$—Fe02—$Fe02^{xi}$	146.41 (4)	$B1^{vii}$—B2—Mo01	65.2 (5)
B1—Fe02—$Fe02^{xi}$	89.6 (2)	B1—B2—Mo01	65.2 (5)
$B2^{viii}$—Fe02—$Fe02^{xi}$	52.33 (6)	$Fe02^{viii}$—B2—Mo01	140.8 (2)
$B2^{vi}$—Fe02—$Fe02^{xi}$	154.4 (4)	Fe02—B2—Mo01	73.56 (19)
$B2^{ix}$—Fe02—$Fe02^{xi}$	49.8 (4)	$Fe02^{xiv}$—B2—Mo01	127.4 (4)
$B2^{x}$—Fe02—$Fe02^{xi}$	99.0 (2)	$Fe02^{vii}$—B2—Mo01	73.56 (19)
B2—Fe02—$Fe02^{xi}$	95.7 (2)	$Fe02^{xvi}$—B2—Mo01	127.4 (4)
$B1^{vi}$—Fe02—$Fe02^{xii}$	89.6 (2)	$B1^{vii}$—B2—$Mo01^{xiv}$	65.2 (5)
B1—Fe02—$Fe02^{xii}$	146.41 (4)	B1—B2—$Mo01^{xiv}$	65.2 (5)
$B2^{viii}$—Fe02—$Fe02^{xii}$	52.33 (6)	$Fe02^{viii}$—B2—$Mo01^{xiv}$	140.8 (2)
$B2^{vi}$—Fe02—$Fe02^{xii}$	95.7 (2)	Fe02—B2—$Mo01^{xiv}$	127.4 (4)
$B2^{ix}$—Fe02—$Fe02^{xii}$	99.0 (2)	$Fe02^{xiv}$—B2—$Mo01^{xiv}$	73.56 (19)
$B2^{x}$—Fe02—$Fe02^{xii}$	49.8 (4)	$Fe02^{vii}$—B2—$Mo01^{xiv}$	127.4 (4)
B2—Fe02—$Fe02^{xii}$	154.4 (4)	$Fe02^{xvi}$—B2—$Mo01^{xiv}$	73.56 (19)
$Fe02^{xi}$—Fe02—$Fe02^{xii}$	69.47 (7)	Mo01—B2—$Mo01^{xiv}$	78.5 (4)

Symmetry codes: (i) −x+2, −y+1, −z+1; (ii) −x+1, −y, −z+1; (iii) x+1, y+1, z; (iv) −x+1, −y+1, −z+1; (v) −x+2, −y, −z+1; (vi) x, y+1, z; (vii) x+1, y, z; (viii) −x+3/2, −y+1/2, −z+1/2; (ix) x−1, y, z; (x) x−1, y+1, z; (xi) −x+1/2, −y+1/2, −z+1/2; (xii) −x+1/2, −y+3/2, −z+1/2; (xiii) −x+3/2, −y+3/2, −z+1/2; (xiv) x, y−1, z; (xv) x−1, y−1, z; (xvi) x+1, y−1, z.

Table S11. Sample data and structure refinement for $FeCoMoB_4$.

Empirical formula	$FeCoMoB_4$
Formula weight	253.96
Temperature	296(2) K
Wavelength	0.71073 Å
Crystal system	Orthorhombic
Space group	*Immm*
	a = 3.0132(3) Å α = 90°
Unit cell dimensions	b = 3.0751(3) Å β = 90°
	c = 12.5432(12) Å γ = 90°
Volume	116.224(19) Å^3
Z	2
Density (calculated)	7.257 g/cm^3
Absorption coefficient	18.114 mm^{-1}
F(000)	230
Crystal size	0.014 x 0.015 x 0.018 mm^3
θ range for data collection	3.52 to 31.26°
Index ranges	-4<=h<=4, -11<=k<=11, -3<=l<=3

Reflections collected	136
Independent reflections	136 [R(sig) = 1.35%]
Coverage of independent reflections	100%
Refinement method	Full-matrix least-squares on F^2
Data / restraints / parameters	136 / 0/ 16
Goodness-of-fit	1.357
Final R indices [>2σ(I)]	R_{obs} = 0.0250, wR_{obs} = 0.0450
R indices [all data]	R_{all} = 0.0254, wR_{all} = 0.0452
Largest diff. peak and hole	0.606 and -0.820 e·Å^{-3}
Max. and min. transmission	0.7900 and 0.6500

$R = \Sigma||F_o|-|F_c|| / \Sigma|F_o|$, $wR = \{\Sigma[w(|F_o|^2 - |F_c|^2)^2] / \Sigma[w(|F_o|^4)]\}^{1/2}$ and calc $w=1/[\sigma^2(Fo^2)+(0.0503P)^2+0.0000P]$ where $P=(Fo^2+2Fc^2)/3$

Table S12. Atomic coordinates and equivalent isotropic atomic displacement parameters (Å^2) for $FeCoMoB_4$.

Label	x	y	z	Occupancy	U_{eq}*
Mo01	0.5	0.5	0.5	1	0.0047(2)
B01	0.0	0.5	0.1498(7)	1	0.0078(15)
B02	0.0	0.0	0.4272(7)	1	0.0082(15)
Co01	0.0	0.5	0.31413(8)	0.5	0.0036(2)
Fe01	0.0	0.5	0.31413(8)	05	0.0036(2)

*U_{eq} is defined as one third of the trace of the orthogonalized U_{ij} tensor.

Table S13. Anisotropic atomic displacement parameters (Å^2) for $FeCoMoB_4$.

Label	U_{11}	U_{22}	U_{33}	U_{12}	U_{13}	U_{23}
Mo01	0.0040(4)	0.0053(4)	0.0048(4)	0	0	0
B01	0.005(3)	0.009(4)	0.009(3)	0	0	0
B02	0.006(3)	0.009(4)	0.009(3)	0	0	0
Co01	0.0042(5)	0.0029(5)	0.0036(4)	0	0	0
Fe01	0.0042(5)	0.0029(5)	0.0036(4)	0	0	0

The anisotropic displacement factor exponent takes the form: $-2\pi^2[h^2a^{*2}U_{11} + \ldots + 2hka^*b^*U_{12}]$.

Table S14. Bond lengths (Å) for $FeCoMoB_4$.

Label	Distances	Label	Distances
Mo01—B02	2.338 (3)	B01—B02^{viii}	1.789 (7)
Mo01—B02^{i}	2.338 (3)	B01—Co01	2.062 (8)
Mo01—B02^{ii}	2.338 (3)	B01—Co01^{xi}	2.1998 (18)
Mo01—B02^{iii}	2.338 (3)	B01—Co01^{xii}	2.1998 (18)
Mo01—B02^{iv}	2.338 (3)	B01—Co01^{xiii}	2.1998 (18)
Mo01—B02^{v}	2.338 (3)	B01—Co01^{viii}	2.1998 (18)
Mo01—B02^{vi}	2.338 (3)	B02—B02^{ii}	1.826 (17)
Mo01—B02^{vii}	2.338 (3)	B02—Co01	2.092 (6)

Mo01—B01^{viii}	2.427 (7)	B02—Co01^{xiv}	2.092 (6)
Mo01—B01^{ix}	2.427 (7)	Co01—Co01^{xii}	2.6873 (11)
Mo01—B01^{x}	2.427 (7)	Co01—Co01^{xi}	2.6873 (11)
Mo01—B01^{xi}	2.427 (7)	Co01—Co01^{xiii}	2.6873 (11)
B01—B02^{xii}	1.789 (7)	Co01—Co01^{viii}	2.6873 (11)

Symmetry codes: (i) −x+2, −y+1, −z+1; (ii) −x+1, −y, −z+1; (iii) x+1, y+1, z; (iv) −x+1, −y+1, −z+1; (v) −x+2, −y, −z+1; (vi) x, y+1, z; (vii) x+1, y, z; (viii) −x+3/2, −y+1/2, −z+1/2; (ix) x, y−1, z; (x) x+1, y−1, z; (xi) −x+1/2, −y+1/2, −z+1/2; (xii) −x+1/2, −y+3/2, −z+1/2; (xiii) −x+3/2, −y+3/2, −z+1/2; (xiv) x−1, y, z; (xv) x−1, y+1, z; (xvi) x−1, y−1, z.

Table S15. Bond angles (°) for FeCoMoB$_4$.

Label	Angles	Label	Angles
B02—Mo01—B02^{i}	180.0	B01^{xii}—B02—B01^{viii}	114.7 (7)
B02—Mo01—B02^{ii}	46.0 (4)	B01^{xii}—B02—B02^{ii}	122.7 (3)
B02^{i}—Mo01—B02^{ii}	134.0 (4)	B01^{viii}—B02—B02^{ii}	122.7 (3)
B02—Mo01—B02^{iii}	134.0 (4)	B01^{xii}—B02—Co01	68.5 (3)
B02^{i}—Mo01—B02^{iii}	46.0 (4)	B01^{viii}—B02—Co01	68.5 (3)
B02^{ii}—Mo01—B02^{iii}	180.0	B02^{ii}—B02—Co01	132.69 (18)
B02—Mo01—B02^{iv}	99.77 (14)	B01^{xii}—B02—Co01^{xiv}	68.5 (3)
B02^{i}—Mo01—B02^{iv}	80.23 (14)	B01^{viii}—B02—Co01^{xiv}	68.5 (3)
B02^{ii}—Mo01—B02^{iv}	82.23 (15)	B02^{ii}—B02—Co01^{xiv}	132.69 (18)
B02^{iii}—Mo01—B02^{iv}	97.77 (15)	Co01—B02—Co01^{xiv}	94.6 (4)
B02—Mo01—B02^{v}	80.23 (14)	B01^{xii}—B02—Mo01	138.87 (9)
B02^{i}—Mo01—B02^{v}	99.77 (14)	B01^{viii}—B02—Mo01	70.62 (17)
B02^{ii}—Mo01—B02^{v}	97.77 (14)	B02^{ii}—B02—Mo01	67.0 (2)
B02^{iii}—Mo01—B02^{v}	82.23 (15)	Co01—B02—Mo01	77.38 (4)
B02^{iv}—Mo01—B02^{v}	180.0	Co01^{xiv}—B02—Mo01	138.42 (17)
B02—Mo01—B02^{vi}	97.77 (15)	B01^{xii}—B02—Mo01^{xvii}	70.62 (17)
B02^{i}—Mo01—B02^{vi}	82.23 (15)	B01^{viii}—B02—Mo01^{xvii}	138.87 (9)
B02^{ii}—Mo01—B02^{vi}	80.23 (14)	B02^{ii}—B02—Mo01^{xvii}	67.02 (19)
B02^{iii}—Mo01—B02^{vi}	99.77 (14)	Co01—B02—Mo01^{xvii}	138.42 (17)
B02^{iv}—Mo01—B02^{vi}	134.0 (4)	Co01^{xiv}—B02—Mo01^{xvii}	77.38 (4)
B02^{v}—Mo01—B02^{vi}	46.0 (4)	Mo01—B02—Mo01^{xvii}	134.0 (4)
B02—Mo01—B02^{vii}	82.23 (15)	B01^{xii}—B02—Mo01^{xviii}	70.62 (17)
B02^{i}—Mo01—B02^{vii}	97.77 (15)	B01^{viii}—B02—Mo01^{xviii}	138.87 (9)
B02^{ii}—Mo01—B02^{vii}	99.77 (14)	B02^{ii}—B02—Mo01^{xviii}	67.0 (2)
B02^{iii}—Mo01—B02^{vii}	80.23 (14)	Co01—B02—Mo01^{xviii}	77.38 (4)
B02^{iv}—Mo01—B02^{vii}	46.0 (4)	Co01^{xiv}—B02—Mo01^{xviii}	138.42 (17)
B02^{v}—Mo01—B02^{vii}	134.0 (4)	Mo01—B02—Mo01^{xviii}	80.23 (14)
B02^{vi}—Mo01—B02^{vii}	180.0	Mo01^{xvii}—B02—Mo01^{xviii}	82.23 (14)
B02—Mo01—B01^{viii}	44.06 (16)	B01^{xii}—B02—Mo01^{xiv}	138.87 (9)

B02^{i}—Mo01—B01^{viii}	135.94 (16)	B01^{viii}—B02—Mo01^{xiv}	70.62 (16)
B02^{ii}—Mo01—B01^{viii}	83.4 (2)	B02^{ii}—B02—Mo01^{xiv}	67.0 (2)
B02^{iii}—Mo01—B01^{viii}	96.6 (2)	Co01—B02—Mo01^{xiv}	138.42 (17)
B02^{iv}—Mo01—B01^{viii}	135.94 (16)	Co01^{xiv}—B02—Mo01^{xiv}	77.38 (4)
B02^{v}—Mo01—B01^{viii}	44.06 (16)	Mo01—B02—Mo01^{xiv}	82.23 (14)
B02^{vi}—Mo01—B01^{viii}	83.4 (2)	Mo01^{xvii}—B02—Mo01^{xiv}	80.23 (14)
B02^{vii}—Mo01—B01^{viii}	96.6 (2)	Mo01^{xviii}—B02—Mo01^{xiv}	134.0 (4)
B02—Mo01—B01^{ix}	135.94 (16)	B01—Co01—B02^{vii}	132.69 (18)
B02^{i}—Mo01—B01^{ix}	44.06 (16)	B01—Co01—B02	132.69 (18)
B02^{ii}—Mo01—B01^{ix}	96.6 (2)	B02^{vii}—Co01—B02	94.6 (4)
B02^{iii}—Mo01—B01^{ix}	83.4 (2)	B01—Co01—B01^{xi}	101.9 (2)
B02^{iv}—Mo01—B01^{ix}	44.06 (16)	B02^{vii}—Co01—B01^{xi}	49.20 (18)
B02^{v}—Mo01—B01^{ix}	135.94 (16)	B02—Co01—B01^{xi}	112.0 (2)
B02^{vi}—Mo01—B01^{ix}	96.6 (2)	B01—Co01—B01^{xii}	101.9 (2)
B02^{vii}—Mo01—B01^{ix}	83.4 (2)	B02^{vii}—Co01—B01^{xii}	112.0 (2)
B01^{viii}—Mo01—B01^{ix}	180.0 (3)	B02—Co01—B01^{xii}	49.20 (18)
B02—Mo01—B01^{x}	83.4 (2)	B01^{xi}—Co01—B01^{xii}	156.2 (4)
B02^{i}—Mo01—B01^{x}	96.6 (2)	B01—Co01—B01^{xiii}	101.9 (2)
B02^{ii}—Mo01—B01^{x}	44.06 (16)	B02^{vii}—Co01—B01^{xiii}	49.20 (18)
B02^{iii}—Mo01—B01^{x}	135.94 (16)	B02—Co01—B01^{xiii}	112.0 (2)
B02^{iv}—Mo01—B01^{x}	96.6 (2)	B01^{xi}—Co01—B01^{xiii}	86.45 (9)
B02^{v}—Mo01—B01^{x}	83.4 (2)	B01^{xii}—Co01—B01^{xiii}	88.69 (9)
B02^{vi}—Mo01—B01^{x}	44.06 (16)	B01—Co01—B01^{viii}	101.9 (2)
B02^{vii}—Mo01—B01^{x}	135.94 (16)	B02^{vii}—Co01—B01^{viii}	112.0 (2)
B01^{viii}—Mo01—B01^{x}	101.4 (3)	B02—Co01—B01^{viii}	49.20 (18)
B01^{ix}—Mo01—B01^{x}	78.6 (3)	B01^{xi}—Co01—B01^{viii}	88.69 (9)
B02—Mo01—B01^{xi}	96.6 (2)	B01^{xii}—Co01—B01^{viii}	86.45 (9)
B02^{i}—Mo01—B01^{xi}	83.4 (2)	B01^{xiii}—Co01—B01^{viii}	156.2 (4)
B02^{ii}—Mo01—B01^{xi}	135.94 (16)	B01—Co01—Co01^{xii}	53.23 (3)
B02^{iii}—Mo01—B01^{xi}	44.06 (16)	B02^{vii}—Co01—Co01^{xii}	145.73 (2)
B02^{iv}—Mo01—B01^{xi}	83.4 (2)	B02—Co01—Co01^{xii}	89.16 (15)
B02^{v}—Mo01—B01^{xi}	96.6 (2)	B01^{xi}—Co01—Co01^{xii}	155.1 (2)
B02^{vi}—Mo01—B01^{xi}	135.94 (16)	B01^{xii}—Co01—Co01^{xii}	48.7 (2)
B02^{vii}—Mo01—B01^{xi}	44.06 (16)	B01^{xiii}—Co01—Co01^{xii}	98.00 (13)
B01^{viii}—Mo01—B01^{xi}	78.6 (3)	B01^{viii}—Co01—Co01^{xii}	96.16 (13)
B01^{ix}—Mo01—B01^{xi}	101.4 (3)	B01—Co01—Co01^{xi}	53.23 (3)
B01^{x}—Mo01—B01^{xi}	180.0	B02^{vii}—Co01—Co01^{xi}	89.16 (15)
B02^{xii}—B01—B02^{viii}	114.7 (7)	B02—Co01—Co01^{xi}	145.73 (2)
B02^{xii}—B01—Co01	122.7 (3)	B01^{xi}—Co01—Co01^{xi}	48.7 (2)
B02^{viii}—B01—Co01	122.7 (3)	B01^{xii}—Co01—Co01^{xi}	155.1 (2)

B02^{xii}—B01—Co01^{xi}	133.5 (2)	B01^{xiii}—Co01—Co01^{xi}	96.16 (13)
B02^{viii}—B01—Co01^{xi}	62.25 (14)	B01^{viii}—Co01—Co01^{xi}	98.00 (13)
Co01—B01—Co01^{xi}	78.1 (2)	Co01^{xii}—Co01—Co01^{xi}	106.46 (6)
B02^{xii}—B01—Co01^{xii}	62.25 (14)	B01—Co01—Co01^{xiii}	53.23 (3)
B02^{viii}—B01—Co01^{xii}	133.5 (2)	B02^{vii}—Co01—Co01^{xiii}	89.16 (14)
Co01—B01—Co01^{xii}	78.1 (2)	B02—Co01—Co01^{xiii}	145.73 (2)
Co01^{xi}—B01—Co01^{xii}	156.2 (4)	B01^{xi}—Co01—Co01^{xiii}	96.16 (13)
B02^{xii}—B01—Co01^{xiii}	62.25 (14)	B01^{xii}—Co01—Co01^{xiii}	98.00 (13)
B02^{viii}—B01—Co01^{xiii}	133.5 (2)	B01^{xiii}—Co01—Co01^{xiii}	48.7 (2)
Co01—B01—Co01^{xiii}	78.1 (2)	B01^{viii}—Co01—Co01^{xiii}	155.1 (2)
Co01^{xi}—B01—Co01^{xiii}	86.45 (9)	Co01^{xii}—Co01—Co01^{xiii}	69.80 (3)
Co01^{xii}—B01—Co01^{xiii}	88.69 (9)	Co01^{xi}—Co01—Co01^{xiii}	68.20 (3)
B02^{xii}—B01—Co01^{viii}	133.5 (2)	B01—Co01—Co01^{viii}	53.23 (3)
B02^{viii}—B01—Co01^{viii}	62.25 (14)	B02^{vii}—Co01—Co01^{viii}	145.73 (2)
Co01—B01—Co01^{viii}	78.1 (2)	B02—Co01—Co01^{viii}	89.16 (14)
Co01^{xi}—B01—Co01^{viii}	88.69 (9)	B01^{xi}—Co01—Co01^{viii}	98.00 (13)
Co01^{xii}—B01—Co01^{viii}	86.45 (9)	B01^{xii}—Co01—Co01^{viii}	96.16 (13)
Co01^{xiii}—B01—Co01^{viii}	156.2 (4)	B01^{xiii}—Co01—Co01^{viii}	155.1 (2)
B02^{xii}—B01—Mo01^{xv}	65.3 (3)	B01^{viii}—Co01—Co01^{viii}	48.7 (2)
B02^{viii}—B01—Mo01^{xv}	65.3 (3)	Co01^{xii}—Co01—Co01^{viii}	68.20 (3)
Co01—B01—Mo01^{xv}	140.70 (13)	Co01^{xi}—Co01—Co01^{viii}	69.80 (3)
Co01^{xi}—B01—Mo01^{xv}	127.0 (2)	Co01^{xiii}—Co01—Co01^{viii}	106.46 (6)
Co01^{xii}—B01—Mo01^{xv}	73.54 (11)	B01—Co01—Mo01	147.129 (11)
Co01^{xiii}—B01—Mo01^{xv}	127.0 (2)	B02^{vii}—Co01—Mo01	55.29 (13)
Co01^{viii}—B01—Mo01^{xv}	73.54 (11)	B02—Co01—Mo01	55.28 (13)
B02^{xii}—B01—Mo01^{xvi}	65.3 (3)	B01^{xi}—Co01—Mo01	57.00 (19)
B02^{viii}—B01—Mo01^{xvi}	65.3 (3)	B01^{xii}—Co01—Mo01	101.5 (2)
Co01—B01—Mo01^{xvi}	140.70 (13)	B01^{xiii}—Co01—Mo01	101.5 (2)
Co01^{xi}—B01—Mo01^{xvi}	73.54 (11)	B01^{viii}—Co01—Mo01	57.00 (19)
Co01^{xii}—B01—Mo01^{xvi}	127.0 (2)	Co01^{xii}—Co01—Mo01	143.81 (3)
Co01^{xiii}—B01—Mo01^{xvi}	73.54 (11)	Co01^{xi}—Co01—Mo01	101.45 (2)
Co01^{viii}—B01—Mo01^{xvi}	127.0 (2)	Co01^{xiii}—Co01—Mo01	143.81 (3)
Mo01^{xv}—B01—Mo01^{xvi}	78.6 (3)	Co01^{viii}—Co01—Mo01	101.45 (2)

Symmetry codes: (i) −x+1, −y+1, −z+1; (ii) −x, −y, −z+1; (iii) x+1, y+1, z; (iv) −x, −y+1, −z+1; (v) x+1, y, z; (vi) −x+1, −y, −z+1; (vii) x, y+1, z; (viii) −x+1/2, −y+1/2, −z+1/2; (ix) x+1/2, y+1/2, z+1/2; (x) x+1/2, y−1/2, z+1/2; (xi) −x+1/2, −y+3/2, −z+1/2; (xii) −x−1/2, −y+1/2, −z+1/2; (xiii) −x−1/2, −y+3/2, −z+1/2; (xiv) x, y−1, z; (xv) x−1/2, y−1/2, z−1/2; (xvi) x−1/2, y+1/2, z−1/2; (xvii) x−1, y−1, z; (xviii) x−1, y, z.

Table S16. A brief summary of Paths of Co_2MoB_4 and Fe_2MoB_4 calculated by Artemis and these parameters are used as model for EXAFS fitting.

Co_2MoB_4			Fe_2MoB_4		
Path	R(Å)	Coordinate Number	Path	R(Å)	Coordinate Number
Co-B	2.07	3	Fe-B	2.11	3
Co-B	2.20	4	Fe-B	2.20	4
Co-Co	2.68	4	Fe-Fe	2.72	4
Co-Mo	2.77	2	Fe-Mo	2.79	2
Co-Co	3.01	2	Fe-Co	2.99	2
Co-Co	3.07	2	Fe-Co	3.10	2
Co-B	3.36	2	Fe-B	3.43	2
Co-B	3.60	2	Fe-B	3.65	8
Co-B	3.67	8	Fe-B	3.75	2
Co-Mo	4.14	4	Fe-Mo	4.17	4

Table S17. Fitting results parameters of Co K-edge EXAFS of Co_2MoB_4 and Fe doped Co_2MoB_4.

Co_2MoB_4				$Co_{1.5}Fe_{0.5}MoB_4$			
Path	R(Å)	CN	σ^2(Å^2)	Path	R(Å)	C N	σ^2(Å^2)
Co-B	2.05	3.81(16)	0.0100(1)	Co-B	2.04	3.25(4)	0.0100(2)
Co-B	2.14	4.06(13)	0.0040(1)	Co-B	2.13	3.04(5)	0.0040(1)
Co-Co	2.66	3.78(6)	0.0061(2)	Co-Co	2.68	3.81(2)	0.0055(1)
Co-Mo	2.77	1.93(6)	0.0099(1)	Co-Mo	2.76	2.02(2)	0.0040(1)
Co-Co	2.96	2.02(6)	0.0040(2)	Co-Co	2.97	2.03(6)	0.0087(2)
Co-Co	3.05	2.08(14)	0.0100(1)	Co-Co	3.03	2.05(6)	0.0069(3)
Co-B	3.76	3.34(9)	0.0040(2)	Co-B	3.34	4.22(7)	0.0078(6)
Co-B	3.51	2.82(11)	0.0045(2)	Co-B	3.67	3.86(9)	0.0071(4)
Co-B	3.98	8.71(17)	0.0040(2)	Co-B	3.93	4.12(6)	0.0040(5)
Co-Mo	4.18	4.04(7)	0.0056(3)	Co-Mo	4.18	4.48(2)	0.0054(1)
$CoFeMoB_4$				$Co_{0.5}Fe_{1.5}MoB_4$			
Path	R(Å)	CN	σ^2(Å^2)	Path	R(Å)	C N	σ^2(Å^2)
Co-B	2.05	2.93(5)	0.0040(1)	Co-B	2.08	2.90(19)	0.0075(3)
Co-B	2.16	3.63(5)	0.0051(1)	Co-B	2.12	2.93(16)	0.0040(2)
Co-Co	2.69	4.12(3)	0.0086(1)	Co-Co	2.69	3.99(8)	0.0061(1)
Co-Mo	2.75	2.06(3)	0.0081(1)	Co-Mo	2.77	2.06(9)	0.0050(1)
Co-Co	2.97	1.94(5)	0.0040(1)	Co-Co	2.97	1.99(31)	0.0056(2)
Co-Co	3.08	1.97(8)	0.0078(1)	Co-Co	3.07	1.78(31)	0.0044(2)
Co-B	3.34	2.90(9)	0.0040(2)	Co-B	3.37	7.60(35)	0.0070(2)
Co-B	3.65	10.18(10)	0.0100(1)	Co-B	3.65	9.72(35)	0.0085(2)
Co-B	3.92	7.70(8)	0.0044(1)	Co-B	3.93	6.65(31)	0.0040(2)
Co-Mo	4.20	2.42(2)	0.0040(1)	Co-Mo	4.19	4.39(11)	0.0069(2)

Table S18. Fitting result parameters of Fe K-edge EXAFS of Fe_2MoB_4 and Co doped Fe_2MoB_4.

**Fe_2MoB_4**				**$Fe_{1.5}Co_{0.5}MoB_4$**			
Path	R(Å)	CN	σ^2(Å^2)	Path	R(Å)	CN	σ^2(Å^2)
Fe-B	2.07	2.27(37)	0.0100(2)	Fe-B	2.11	2.49(7)	0.0050(1)
Fe-B	2.16	4.06(41)	0.0047(1)	Fe-B	2.13	3.79(7)	0.0040(1)
Fe-Fe	2.70	4.24(20)	0.0100(1)	Fe-Fe	2.70	3.99(7)	0.0069(1)
Fe-Mo	2.75	2.07(11)	0.0093(2)	Fe-Mo	2.75	1.99(5)	0.0068(1)
Fe-Fe	2.99	1.97(9)	0.0040(1)	Fe-Fe	2.98	1.95(11)	0.0040(1)
Fe-Fe	3.11	1.84(34)	0.0100(3)	Fe-Fe	3.09	2.00(12)	0.0099(1)
Fe-B	3.38	3.05(45)	0.0040(4)	Fe-B	3.36	3.25(15)	0.0040(3)
Fe-B	3.64	7.53(46)	0.0043(1)	Fe-B	3.62	10.34(23)	0.0100(1)
Fe-B	3.93	7.48(51)	0.0040(2)	Fe-B	3.91	7.62(22)	0.0061(1)
Fe-Mo	4.20	2.30(14)	0.0060(1)	Fe-Mo	4.21	4.30(7)	0.0064(1)
**$FeCoMoB_4$**				**$Fe_{0.5}Co_{1.5}MoB_4$**			
Path	R(Å)	CN	σ^2(Å^2)	Path	R(Å)	CN	σ^2(Å^2)
Fe-B	2.05	2.90(8)	0.0100(1)	Fe-B	2.10	2.36(3)	0.0062(1)
Fe-B	2.13	4.06(12)	0.0040(1)	Fe-B	2.13	3.62(3)	0.0040(1)
Fe-Fe	2.67	4.02(8)	0.0099(1)	Fe-Fe	2.68	4.00(2)	0.0050(1)
Fe-Mo	2.71	1.96(4)	0.0100(1)	Fe-Mo	2.76	3.42(3)	0.0098(1)
Fe-Fe	2.96	2.02(3)	0.0040(1)	Fe-Fe	2.96	1.76(2)	0.0040(1)
Fe-Fe	3.11	2.03(12)	0.0100(1)	Fe-Fe	3.07	1.92(4)	0.0100(1)
Fe-B	3.38	4.53(25)	0.0100(2)	Fe-B	3.68	5.88(8)	0.0100(3)
Fe-B	3.55	6.47(17)	0.0082(1)	Fe-B	3.67	2.07(7)	0.0100(8)
Fe-B	3.81	5.44(22)	0.0100(1)	Fe-B	3.95	8.15(7)	0.0040(1)
Fe-Mo	4.17	3.08(4)	0.0061(1)	Fe-Mo	4.19	3.84(2)	0.0040(1)

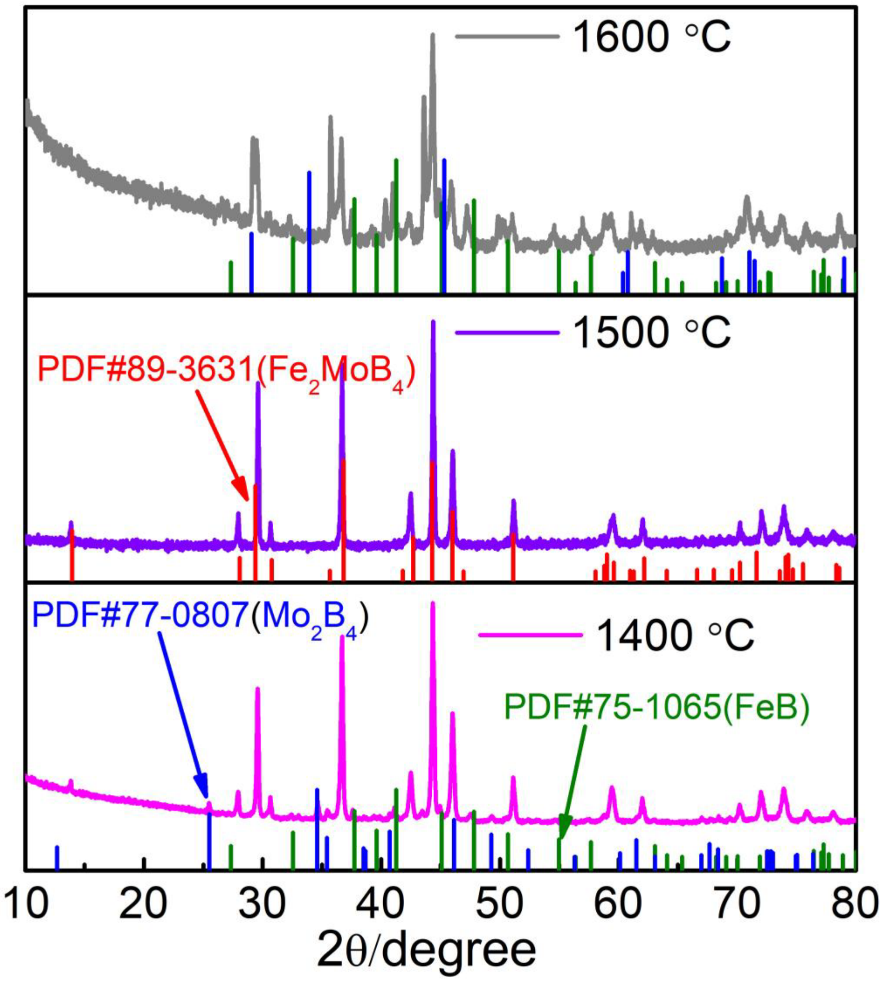

Figure S1. XRD pattern of Fe_2MoB_4 synthesized at 1400°C, 1500°C, 1600°C.

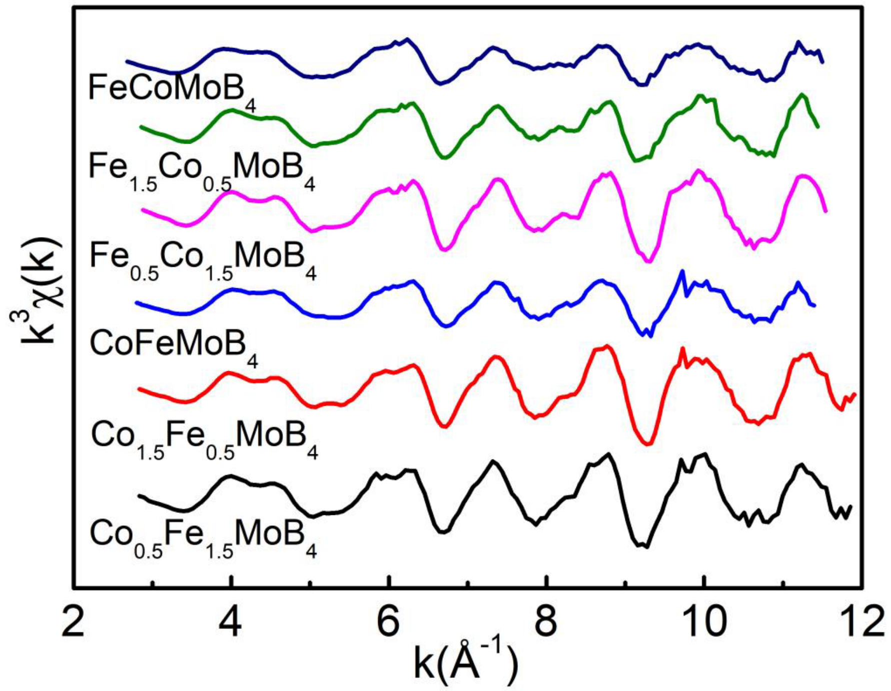

Figure S2. k^3 weighted $\chi(k)$ spectra of Co, Fe co-doped samples, the first element is the absorbing element.

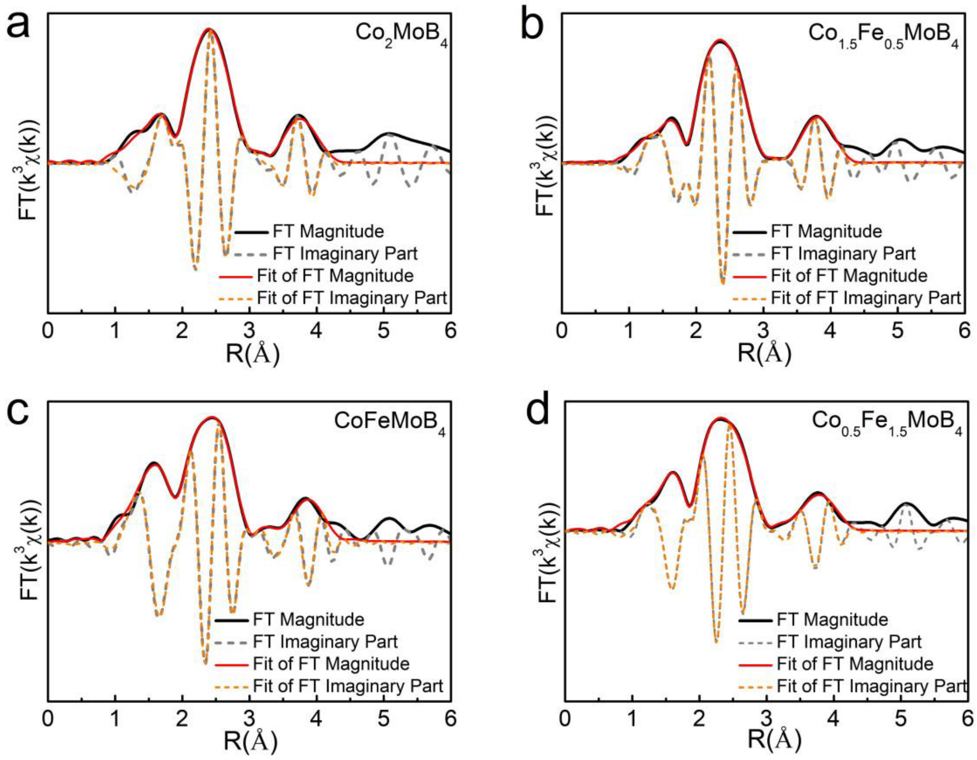

Figure S3. (a), (b) and (c) are Fourier transformed $k^3\chi(k)$ oscillations measured at Co K-edge fitted by structure models of Co_2MoB_4; (d) Fourier transformed $k^3\chi(k)$ oscillations measured at Co K-edge fitted by structure models of Fe_2MoB_4.

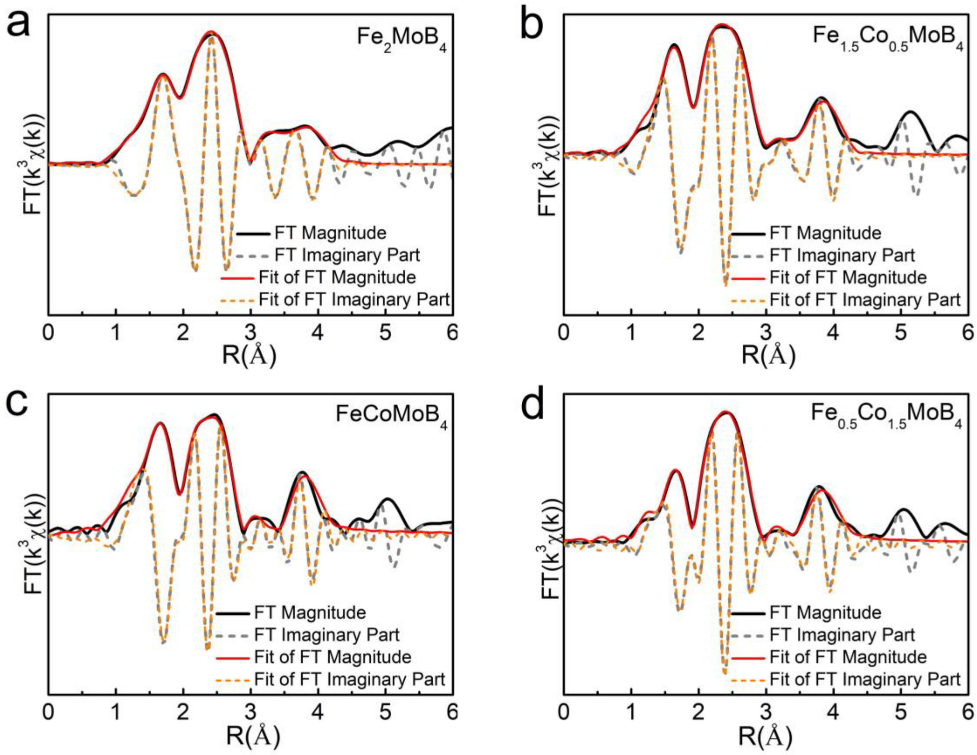

Figure S4. (a), (b) and (c) are Fourier transformed $k^3\chi(k)$ oscillations measured at Fe K-edge fitted by structure models of Fe_2MoB_4; (d) Fourier transformed $k^3\chi(k)$ oscillations measured at Fe K-edge fitted by structure models of Co_2MoB_4.

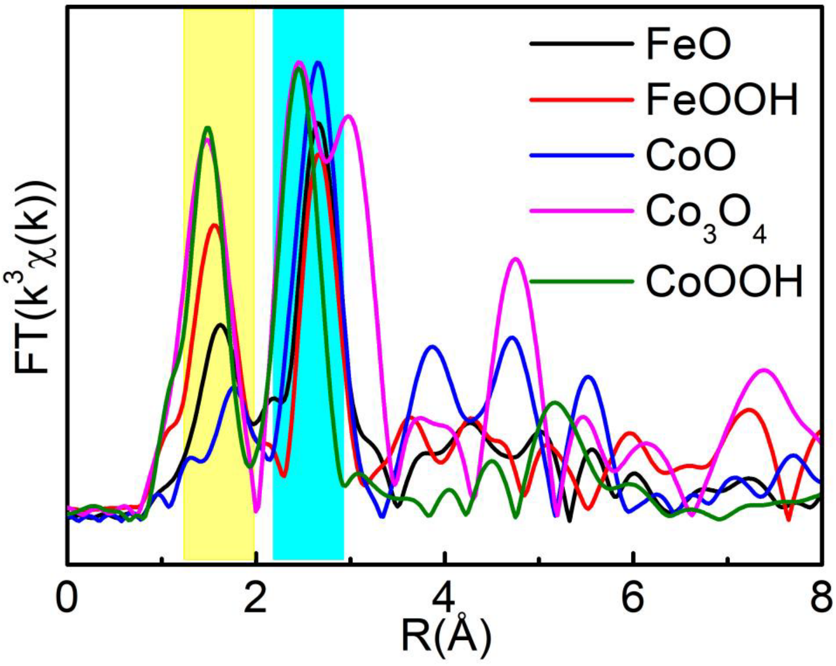

Figure S5. Fourier transformed k^3 weight $\chi(k)$ of Co and Fe related oxides/oxyhydroxides.

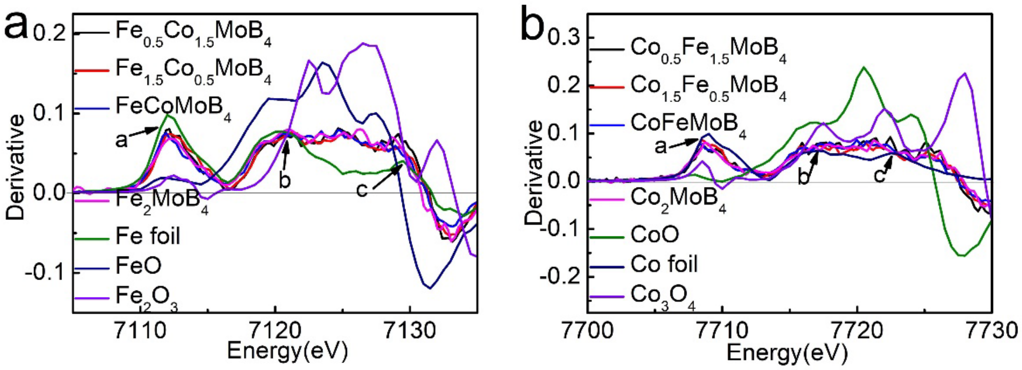

Figure S6. (a) First derivative curves of different samples at Fe K edge XANES; (b) First derivative curves of different samples at Co K edge XANES;

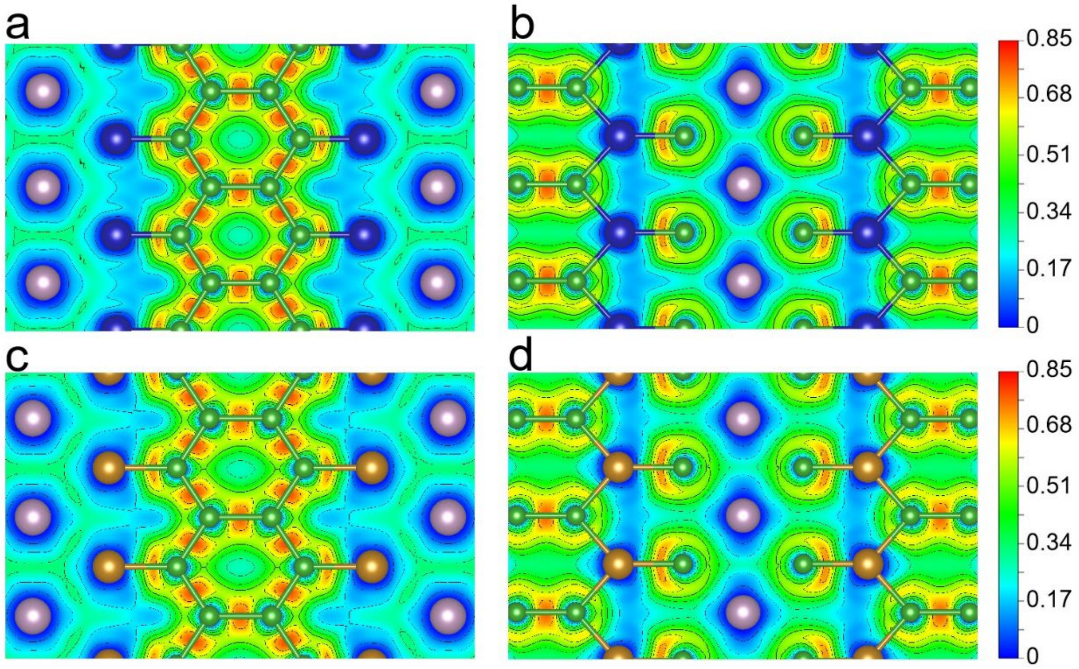

Figure S7. (a-b) The electron localization function (ELF) on the ac and *bc* plane for Co_2MoB_4. The pink, blue and green spheres represent Mo atoms, Co atoms and B atoms, respectively; (c-d) The electron localization function (ELF) on the ac and *bc* plane for Fe_2MoB_4. The pink, brown and green spheres represent Mo atoms, Fe atoms and B atoms, respectively.

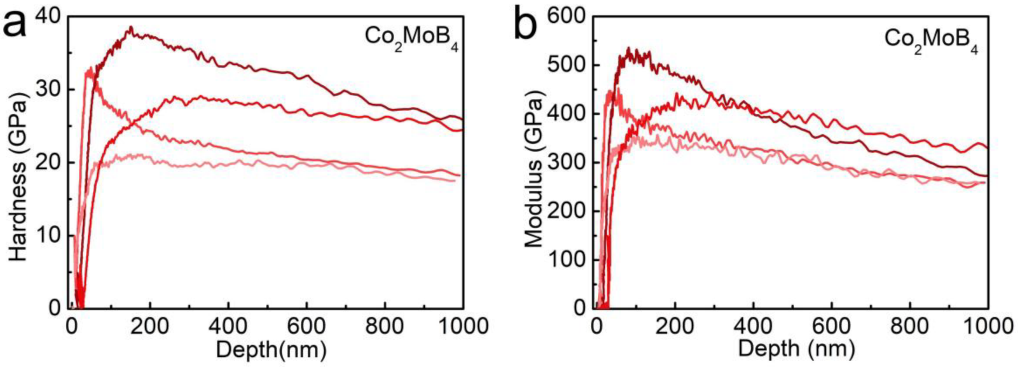

Figure S8. Curves of relationship between hardness/modulus and depth for Co_2MoB_4.

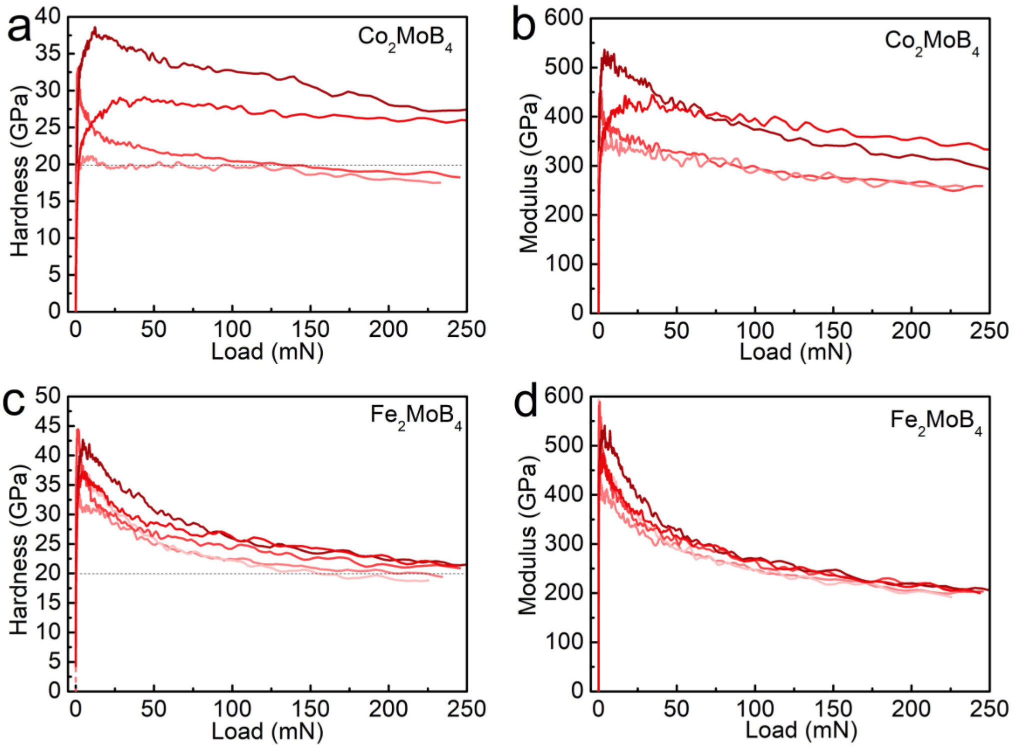

Figure S9. Curves of relationship between hardness/modulus and load for Co_2MoB_4 and Fe_2MoB_4.

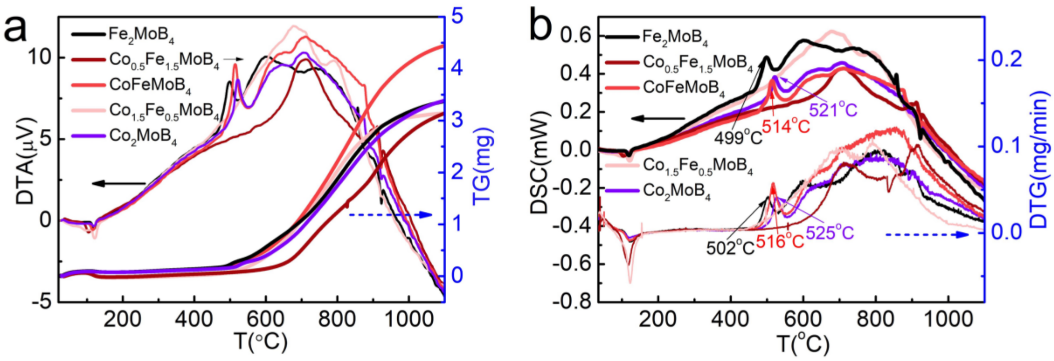

Figure S10. (a) DTA-TG curves of all the samples; (b) DSC-DTG curves of all the sample.
